# CRISPR screens uncover protective effect of PSTK as a regulator of chemotherapy-induced ferroptosis in hepatocellular carcinoma

**DOI:** 10.1186/s12943-021-01466-9

**Published:** 2022-01-04

**Authors:** Yiran Chen, Li Li, Jie Lan, Yang Cui, Xiaosong Rao, Jing Zhao, Tao Xing, Gaoda Ju, Guangtao Song, Jizhong Lou, Jun Liang

**Affiliations:** 1 Key Laboratory of Carcinogenesis and Translational Research (Ministry of Education), Department of Oncology, Peking University Cancer Hospital and Institute, 1 Life Park Road, Life Science Park of Zhongguancun, Changping, Beijing, 102206 P.R. China; 2 Department of Oncology, Peking University International Hospital, Beijing, 102206 China; 3 Laboratory of RNA Biology, CAS Center for Excellence in Biomacromolecules, Institute of Biophysics, Chinese Academy of Sciences, Beijing, 100101 China; 4 Department of Pathology, Boao Evergrande International Hospital, Qionghai, 571400 Hainan China; 5 Department of Pathology, Peking University International Hospital, Beijing, 102206 China; 6 Department of Medical Oncology, The Affiliated Hospital of Qingdao University, Qingdao University, Qingdao, 266000 China; 7 Bioland Laboratory (Guangzhou Regenerative Medicine and Health Guangdong Laboratory), Guangzhou, 510005 China

**Keywords:** Hepatocellular carcinoma, CRISPR library screening, PSTK, Ferroptosis

## Abstract

**Background:**

Hepatocellular carcinoma (HCC) is among the most common forms of cancer and is associated with poor patient outcomes. The emergence of therapeutic resistance has hampered the efficacy of targeted treatments employed to treat HCC patients to date. In this study, we conducted a series of CRISPR/Cas9 screens to identify genes associated with synthetic lethality capable of improving HCC patient clinical responses.

**Methods:**

CRISPR-based loss-of-function genetic screens were used to target 18,053 protein-coding genes in HCC cells to identify chemotherapy-related synthetic lethal genes in these cells. Synergistic effects were analyzed through *in vitro* and *in vivo* analyses, while related mechanisms were explored through RNA-seq and metabolomics analyses. Potential inhibitors of identified genetic targets were selected through high-throughput virtual screening.

**Results:**

The inhibition of phosphoseryl-tRNA kinase (PSTK) was found to increase HCC cell sensitivity to chemotherapeutic treatment. PSTK was associated with the suppression of chemotherapy-induced ferroptosis in HCC cells, and the depletion of PSTK resulted in the inactivation of glutathione peroxidative 4 (GPX4) and the disruption of glutathione (GSH) metabolism owing to the inhibition of selenocysteine and cysteine synthesis, thus enhancing the induction of ferroptosis upon targeted chemotherapeutic treatment. Punicalin, an agent used to treat hepatitis B virus (HBV), was identified as a possible PSTK inhibitor that exhibited synergistic efficacy when applied together with Sorafenib to treat HCC in vitro and in vivo.

**Conclusions:**

These results highlight a key role for PSTK as a mediator of resistance to targeted therapeutic treatment in HCC cells that functions by suppressing ferroptotic induction. PSTK inhibitors may thus represent ideal candidates for overcoming drug resistance in HCC.

**Supplementary Information:**

The online version contains supplementary material available at 10.1186/s12943-021-01466-9.

## Background

Hepatocellular carcinoma (HCC) is a highly deadly form of cancer that is becoming increasingly present throughout the world [1]. Sorafenib was the first approved multi-kinase inhibitor for patients with advanced HCC^2^. However, the objective response rate (ORR) of HCC patients to Sorafenib is under 10%, and this inhibitor cannot prevent advanced HCC progression owing to the emergence of drug resistance [2]. HCC tumor development is associated with the progressive acquisition of mutations in genes encoding many cell cycle related proteins [3]. Consistently, small molecule drugs targeting cyclin-dependent kinases (CDKs) have been employed to suppress such tumor development [4]. For example, Palbociclib (PD0332991) is a specific CDK4/6 inhibitor developed by Pfizer that was shown to be active in advanced HCC patients following failed first-line Sorafenib treatment [5]. A further understanding of the mechanisms governing HCC tumor cell resistance to these targeted therapy agents is vital to guide further efforts to sensitize HCC cells to these potent drugs.

Ferroptosis is a form of programmed cell death that is distinct from necrosis, apoptosis, and autophagy with respect to associated genetic processes, biochemical activities, and morphological characteristics [6]. Ferroptosis is characterized by the accumulation of lipid peroxidation products and oxidized phospholipids in an iron-dependent manner [7]. As cancer cells utilize higher levels of iron than normal cells as a means of facilitating their more aggressive growth, tumors are more sensitive to ferroptosis-inducing agents [8]. Such ferroptosis inducers have thus been proposed as promising tools for the treatment of therapy-resistant tumors, particularly in HCC [9]. Sorafenib is among the most important drugs approved for the treatment of HCC, and it can induce ferroptosis via inhibiting the function of the critical System Xc-, which is an antiporter for cystine-glutamate exchange. As such, efforts to increase HCC cell susceptibility to ferroptosis would be expected to sensitize these cells to Sorafenib [10].

CRISPR/Cas9 is a powerful gene-editing technology that has been used to conduct a growing number of high-throughput screens [11]. CRISPR/Cas9 based library screen system can be used to identify genes whose inhibition increases or decreases the therapeutic efficacy of anticancer drugs [12–14]. Several CRISPR/Cas9 screens have been performed to identify novel HCC targets [15–18]. To extend these analyses, we herein performed a series of CRISPR/Cas9 knockout screens using HCC cells treated with Tyrosine Kinase Inhibitors (TKIs), CDK4/6 inhibitors, or Erastin (A typical ferroptosis inducer) as a means of evaluating the mechanisms driving HCC cell resistance to these therapeutic agents. Through this approach, we identified phosphoseryl-tRNA kinase (PSTK), an essential RNA-dependent kinase [19], as a critical mediator of HCC cell resistance to targeted therapy. PSTK is a key mediator of selenocysteine biosynthesis [20], which forms the activity center in selenoproteins [21]. Our data confirmed an essential role for PSTK in the regulation of ferroptosis-related resistance to chemotherapies in HCC cells. Specifically, we found that PSTK was able to protect against ferroptotic induction by maintaining glutathione peroxidase 4 (GPX4) activity and by promoting glutathione (GSH) metabolism and folate biosynthesis. As such, our findings suggest that targeting PSTK may represent a viable approach to overcoming chemotherapy resistance in HCC via the induction of ferroptosis.

## Methods

### CRISPR/Cas9 knockout library screening

**Fig. 1:**
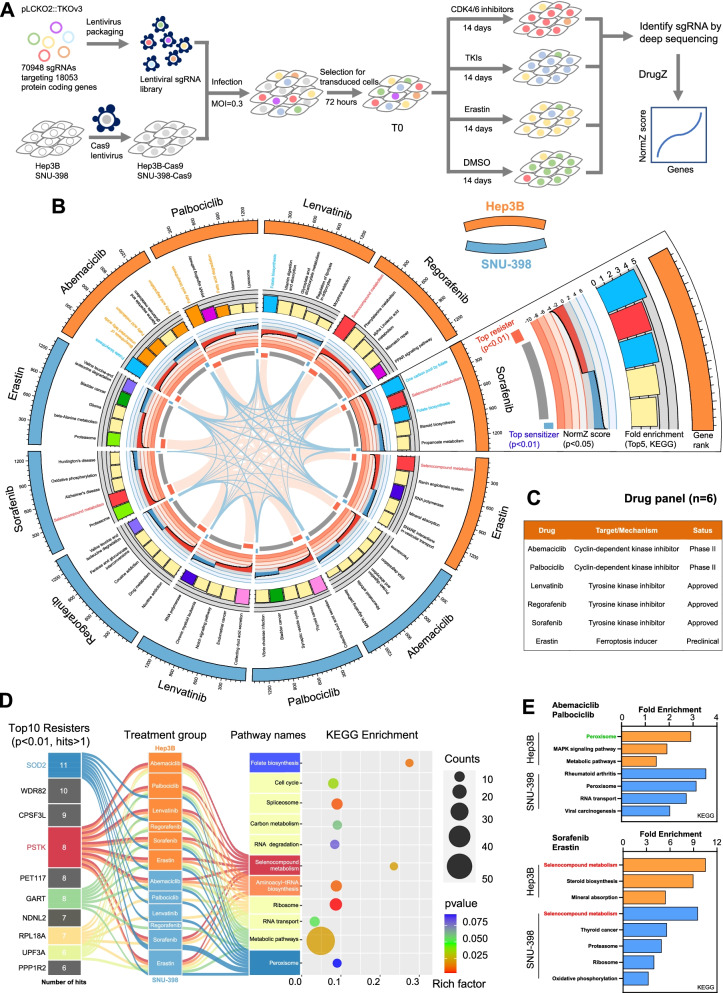
CRISPR/Cas9 screening identifies driver genes associated with HCC cell resistance to targeted therapy. **A** Schematic overview of the CRISPR/Cas9 knockout library screening strategy used in this study. **B** Circos plots displaying CRISPR screen results. Significant screen hits are ranked on the outermost rim (p<0.05) from most sensitizing to most resistance-associated. Histograms and corresponding annotations summarize enriched KEGG pathways for ranked genes, with similar pathways being marked using the same color. Line plots display NormZ scores for ranked genes, with the innermost rim demonstrating the top resister hits (red, p<0.01), top sensitizer hits (blue, p<0.01), and inner (grey). Gene-gene relationships between different treatment groups and different cell lines are indicated with links. **C** A drug panel with information regarding the names, mechanisms of action, and clinical status of drugs used in this study. **D** A Sankey plot demonstrating the relationships among top treatment resistance-related genes, treatment groups, and top KEGG enrichment pathways. Top resistance genes (p<0.01) detected in at least two treatment groups (>1 hits) were used for KEGG enrichment analyses. **E** Subgroup KEGG enrichment analyses for significant hits derived from screening results (upper: the intersection between the Abemaciclib and Palbociclib treatment groups; lower: the intersection between the Sorafenib and Erastin treatment groups)

The Toronto human knockout pooled library (TKOv3) was a gift from Jason Moffat [22] (Addgene #125517). This library contains 70,948 sgRNAs targeting 18,053 protein-coding genes. The general knockout screening workflow for this study is outlined in Fig.1A. Library amplification and virus preparation were performed as per the Moffat Lab protocols. Briefly, HCC cell lines stably expressing Cas9 were established by lentivirally transducing these cells with the Cas9 coding sequence. The resultant Hep3B-Cas9 cells or SNU-398-Cas9 cells were transduced with the TKOv3 library at a low MOI (0.2-0.3) for 24 h, after which they were cultured in fresh DMEM containing puromycin (3 μg/mL) for 72 h. Cells were then separated into eight groups (~1000-fold library coverage in each group). One group of these transfected cells was harvested at baseline prior to treatment (T0), while cells in the other groups were treated with appropriate inhibitors (IC_20_) or vehicle for 14 days. PCR was used to amplify sgRNA barcodes, and deep sequencing was then performed to assess the relative enrichment or dropout of different sgRNA sequences. The abundance of sgRNAs was counted by Bowtie2 and the synergistic/suppressor chemical genetic interactions were analyzed by DrugZ algorithm and NormZ scores [12].

### Establishment of PSTK knockout cells

A dual-sgRNA CRISPR/Cas9-mediated gene deletion strategy was used to improve the efficiency of PSTK knockout by integrating two PSTK-specific sgRNA sequences into one vector (Fig. S1A). Cas9-expressing HCC cell lines were then transduced with lentiviral particles encoding this PSTK-sgRNA1-sgRNA2 construct for 48 h, followed by culture in puromycin-containing media to select for successfully transduced cells. Western blotting was performed to assess knockout efficiency. The sgRNA sequences were listed as follows: PSTK-sgRNA1: 5’-TGCCCCGGCGAGAAACGCGT-3’, PSTK-sgRNA2: 5’- TTTCCGCGGCCCGTCGCTGC-3’.

### High-throughout virtual screen for potential PSTK inhibitors

To identify potential PSTK inhibitors, Autodock Vina was used for structure based virtual screen (VS). TargetMol database contains 8380 compounds was used as VS library. Flowchart of VS was shown in Fig. 7A. The detail methods of VS were described in the supplementary method.

### Xenograft

Animal experiments were carried out in accordance with Institute of Biophysics, Chinese Academy of Science’s Policy on Care and Use of Laboratory Animal. The methods used in the animal studies are described in the supplementary method.

### Patient samples and human ethics approval

We collected HCC specimens and paired normal liver specimens from 50 patients who underwent surgery. This study was approved by the Ethics Committee of Peking University International Hospital (Beijing, China). Patient details are available in Table S1.

## Results

### CRISPR/Cas9 based screening identifies PSTK as a critical mediator of HCC cell resistance to chemotherapy

**Fig. 2:**
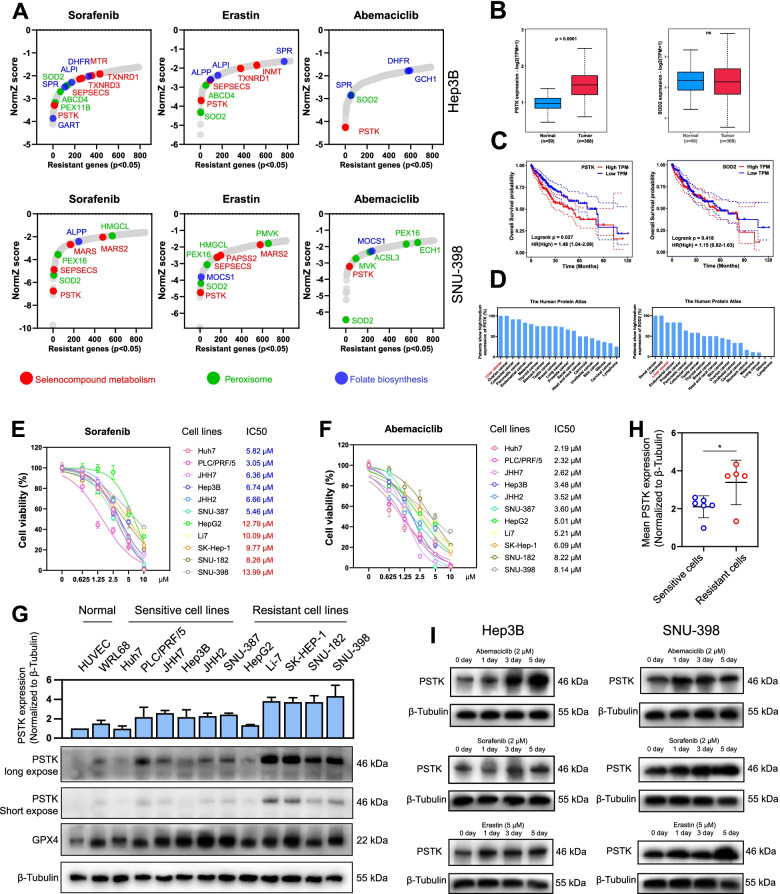
PSTK is a critical gene associated with targeted therapy resistance in HCC cells. **A** Genes associated with selenocompound metabolism, peroxisomes, and folate biosynthesis were strongly depleted, as indicated by the colored dots. PSTK and SOD2 were the most significantly depleted gene hits in most treatment groups. **B** The PSTK/SOD2 mRNA levels in HCC samples and normal liver tissue samples from the TCGA cohort, as analyzed using Student’s t-tests. **C** Kaplan-Meier curves demonstrating that higher PSTK mRNA levels were correlated with a poor prognosis for HCC patients in the TCGA cohort. **D** The percentages of PSTK/SOD2 high/medium expression in comprehensive tumor samples. **E-F** HCC cells viability following after treatment for 48 h with increasing Abemaciclib/Sorafenib concentrations. HCC cell lines were defined as sensitive cell lines (red) or resistant cell lines (blue) according to the median IC_50_ value of Sorafenib. **G-H** Immunoblots (n=3) for the defined sensitive and resistant HCC cell lines, showing expression levels of PSTK, GPX4 and β-Tubulin. *p<0.05. **G** Abemaciclib (2 μM), Sorafenib (2 μM) and Erastin (5 μM) treatments resulted in a time-dependent increase in PSTK protein expression over a 0 to 5 days period

Normalized Z-scores (NormZ) for genes measured using DrugZ were utilized as a means of identifying genes associated with sensitization or resistance to the indicated drug treatments. Cells carrying sgRNAs specific for resistance genes (resisters) were negatively selected in the presence of inhibitors such that they were less abundant in the final pool (NormZ<0), whereas cells carrying sgRNAs specific for sensitizer genes were positively selected such that they were more abundant in the final pool (NormZ>0). Connections among cell lines and targeted therapies were identified by using a circus plot to represent these CRISPR screening results (NormZ scores, p<0.05) (Fig. 1B). In this plot, segments connected by links represent the shared top resisters (p<0.01) or top sensitizers (p<0.01). There were far more links connecting different resistance genes in this analysis as compared to the number of different sensitizer genes (Fig. 1B). For details regarding the drugs used in this study, see Fig. 1C. Of the six inhibitors used in the present screens, Abemaciclib exhibited the most robust cytotoxic efficacy (Fig. S2). KEGG enrichment analysis results corresponding to these different treatment conditions are also listed in the indicated plot segments together with common gene sets, which were marked using the same colors. KEGG enrichment analyses were performed for top resistance genes included in these linkages (p<0.01, hits>1), and the results were ranked by the number of hits. Sankey plots were additionally used to highlight the relationship between top resisters, treatment groups, and KEGG term enrichment (Fig. 1D). These analyses indicated that genes from pathways associated with the selenocompound metabolism, folate biosynthesis, and peroxisome pathways were significant mediators of HCC cell chemotherapy resistance (Fig. 1D). Subgroup analyses were performed according to inhibitor targets and cell lines (Fig. 1E). Significant genes (p<0.05) were enriched in the peroxisome pathway in the CDK4/6 inhibitor-treated cell groups, while Sorafenib and Erastin, which are able to induce ferroptosis, were associated with enriched genes related to selenocompound metabolism (Fig. 1E). Phosphoseryl-tRNA Kinase (PSTK), which is an activated intermediate of selenocysteine biosynthesis that specifically phosphorylates seryl-tRNA (Sec) to O-phosphoseryl-tRNA (Sec) [20], were commonly identified as top resister upon a majority of targeted therapy treatment groups (Fig. 2A and Fig. S3). Manganese Superoxide Dismutase (SOD2 or MnSOD) were also identified as the top negatively selected genes upon screening (Fig. 2A and Fig. S3).

### PSTK promotes enhanced chemotherapy resistance in HCC cells

Analyses of the TCGA database indicated that PSTK is commonly upregulated in HCC patient tumor tissue samples as compared to normal control tissues, and patients with higher levels of PSTK expression exhibit a worse prognosis (Fig. 2B-C). In contrast, no differences in SOD2 expression were detected in HCC patient tissues in this database, nor were SOD2 expression levels predictive of patient survival outcomes (Fig. 2B-C). Analyses of data in The Human Protein Atlas further revealed that both PSTK and SOD2 were expressed at high levels in HCC relative to other tumor types (Fig. 2D). In a pan-cancer TCGA analysis, PSTK was also markedly upregulated in HCC (Fig. S4A). Protein-Protein interaction networks and associated functional enrichment analyses performed using the STRING databases confirmed that PSTK is associated with selenocysteine biosynthesis (Fig. S4B). HCC tumors also exhibited the overexpression of PSTK-interacting genes, and such expression was also associated with worse patient outcomes (Fig. S4C). Moreover, PSTK expression is higher in poorly differentiated HCC samples than well differentiated HCC (Fig. S4D) and PSTK expression is higher in HCC samples with TP53 mutation (Fig. S4E). We evaluated the activity of Sorafenib and Abemaciclib across 11 HCC cell lines as determined via CCK-8 assay (Fig. 2E-F). The median of IC_50_ values of Sorafenib was set as the cutoff to group therapy sensitive cell lines (6/11 cell lines) and therapy resistant cell lines (5/11 cell lines) (Fig. 2E). We confirmed PSTK expression levels in 11 HCC cell lines and two normal cell lines by Western blotting (Fig. 2G). Mean PSTK expressions in Sorafenib resistant cell lines are compared to that in Sorafenib-sensitive cell lines (Fig. 2H). We additionally observed progressive increases in PSTK expression following targeted therapies (Fig. 2I). These findings suggested that PSTK is a specific target that can enable HCC cells to better resist targeted therapies.

PSTK knockout increases HCC cell sensitivity to chemotherapies

**Fig. 3:**
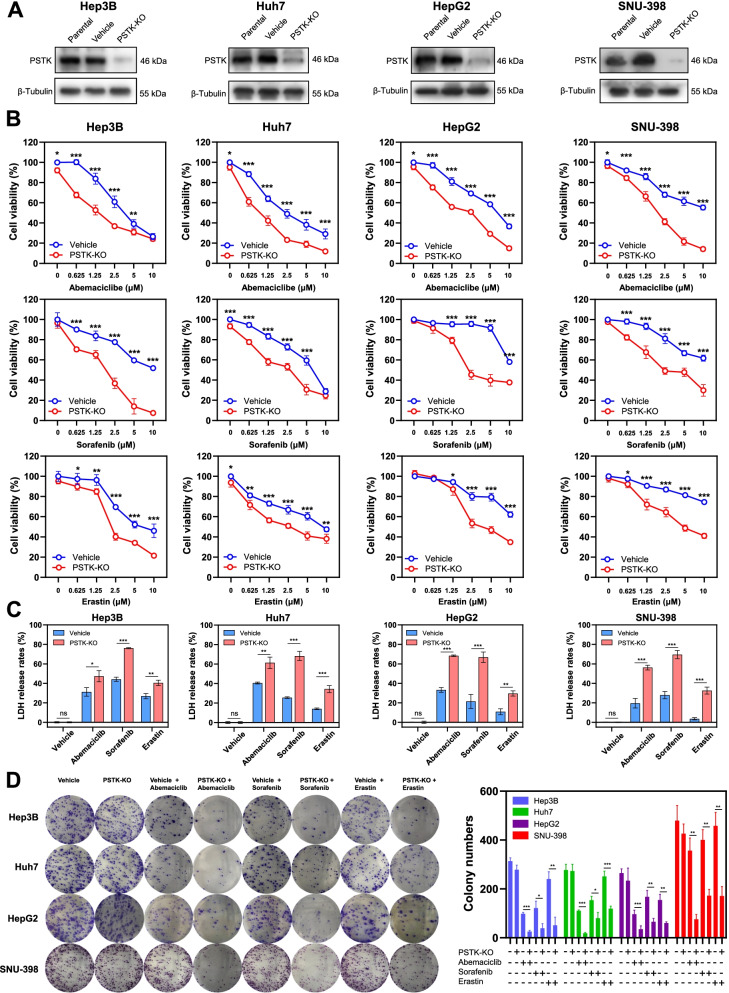
PSTK knockout sensitizes HCC cells to targeted therapy *in vitro*. **A** PSTK was knocked out in Hep3B, Huh7, HepG2, and SNU-398 cells, with Western blotting being used to confirm the efficiency of knockout. **B** PSTK-KO cells and control sgRNA transfected cells were treated for 48 h with a range of Abemaciclib/Sorafenib/Erastin doses, after which a CCK-8 assay was used to assess cell viability relative to vehicle-treated cells (n=5). **C** PSTK-KO cells and control sgRNA-transfected cells were treated for 48 h with Abemaciclib (5 μM), Sorafenib (5 μM), or Erastin (10 μM), after which an LDH assays were used to assess cell death (n=3). **D** Colony formation assays were conducted by plating PSTK-KO or control cell lines (800 cells/well) and treating them with Abemaciclib (2 μM), Sorafenib (2 μM), Erastin (5 μM), or vehicle control for 10 days (n=3). *p<0.05; **p<0.01; ***p<0.001; Student’s t-test

**Fig. 4:**
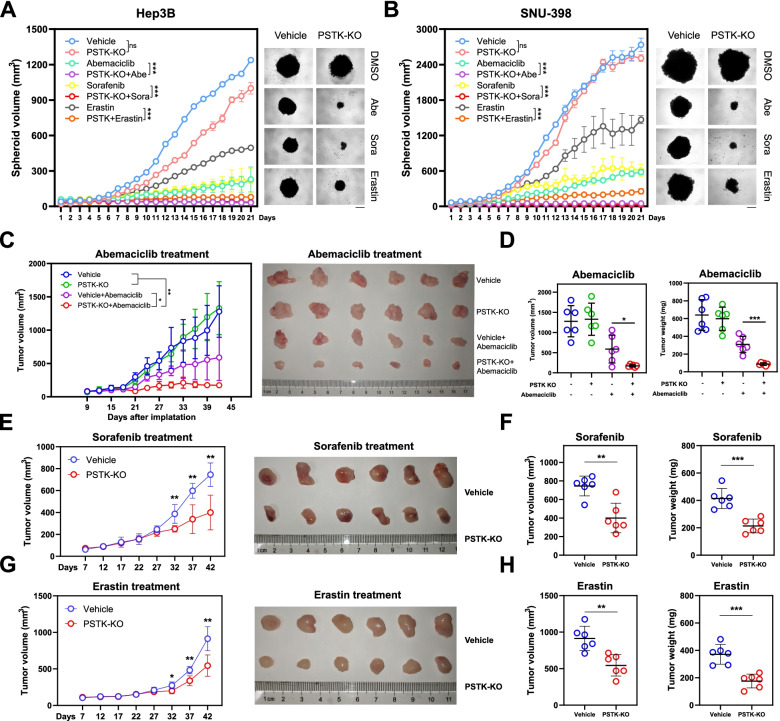
PSTK knockout increases the sensitivity of HCC to therapeutic agents in spheroid cultures and *in vivo.* **A-B** Longitudinal changes in the volume of spheroids prepared from PSTK-KO and control cells under the indicated treatments over a 21-day period (n=6). Right: the final spheroids in the indicated groups. Scale bar: 500 μm. **C** Changes in tumor volumes over time for mice implanted with PSTK-KO Hep3B cells and control cells and treated with Abemacicilb or vehicle. **D** Measures of tumor volume and tumor weight values in mice bearing PSTK-KO Hep3B tumor xenografts following treatment with Abemaciclib or vehicle control. **E-F** Longitudinal changes in tumor volume and final tumor weight values for xenograft-bearing mice treated with Sorafenib. **G-H** Longitudinal changes in tumor volume and final tumor weight values in xenograft-bearing mice treated with Erastin. *p<0.05; **p<0.01; ***p<0.001; Student’s t-test

To confirm the results of our screen, we next generated PSTK-KO cells by transducing Cas9-expressing HCC cells with lentiviral particles encoding a dual-sgRNA plasmid construct. This approach achieved efficient PSTK knockout in four HCC cell lines (Fig. 3A). In short-term assays, PSTK knockout was associated with significantly enhanced Abemaciclib, Sorafenib, and Erastin suppressive activity (Fig. 3B-C). However, PSTK knockout did not induce significant cellular apoptosis or changes in cell cycle proportion (Fig. S5A-B). We transfected a PSTK overexpression plasmid into Sorafenib sensitive cells (Hep3B and Huh7), and this significantly increased Sorafenib resistance (Fig. S5C). In long-term assays, PSTK knockout was associated with a slight reduction in HCC cell growth when plated at low confluency and cultured for 10 days (Fig. S5D). In a colony formation assay, PSTK-KO cells exhibited enhanced Abemaciclib, Sorafenib, and Erastin sensitivity over a 10-day period treatment (Fig. 3D). We found that PSTK slightly inhibited the growth of HCC spheroids cultured ultra-low attachment round-bottom 96-well plate (Fig. S5E-F), and significantly increased the sensitivity of these spheroids to targeted therapies (Fig. 4A-B). Next, we subcutaneously implanted Nod-SCID mice with Hep3B-PSTK-KO cells and Hep3B-vehicle cells. When tumors were palpable, these mice were intraperitoneally treated with Abemaciclib or vehicle control. Significantly enhanced growth inhibition was evident when mice bearing PSTK-KO tumors were treated with Abemaciclib as compared to vehicle, whereas the knockout of PSTK did not significantly suppress the growth of untreated tumors (Fig. 4C-D). PSTK depletion was also associated with significant increases in Hep3B cell sensitivity to Sorafenib or Erastin treatment (Fig. 4E-H). The body weights of mice in the drug treatment groups did not differ significantly from the control groups. Together, these results confirmed that PSTK knockout was sufficient to increase HCC cell sensitivity to Abemaciclib, Sorafenib, and Erastin.

### PSTK regulates ferroptosis-related gene expression

**Fig. 5:**
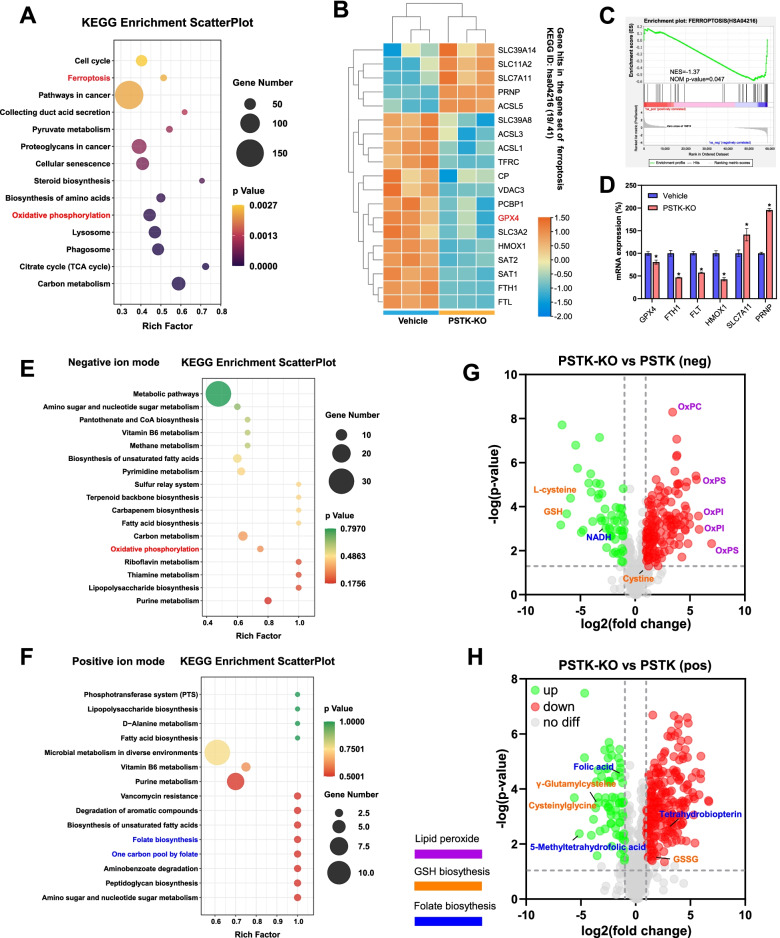
Comprehensive metabolomics and RNA-seq analyses identify PSTK as a mediator of ferroptosis resistance. **A** KEGG enrichment analyses of genes differentially expressed following PSTK knockout. **B** Heatmaps demonstrating gene hits in the ferroptosis gene set. **C** Gene Set Enrichment Analysis demonstrating that ferroptosis-related genes were significantly enriched following PSTK knockout. **D** The mRNA expression levels of key ferroptosis-related genes in PSTK-KO and control cells. *p<0.05; Student’s t-test. **E** KEGG enrichment analysis of differentially abundant metabolites identified in negative ion mode. **F** KEGG enrichment analysis of differentially abundant metabolites identified in positive ion mode. **G-H** Volcano plots demonstrating altered metabolite levels. Metabolites associated with GSH metabolism and folate biosynthesis were significantly downregulated, while oxidized phospholipids were significantly upregulated

Next, we performed an RNA-seq analysis of the effects of PSTK-KO on global transcriptional patterns in Hep3B cells. Differentially expressed genes were subjected to KEGG enrichment analyses (Fig. 5A), which revealed the significant differential expression of ferroptosis-related genes following PSTK knockout (Fig. 5A-B). GSEA analyses revealed the ferroptosis gene set to be negatively enriched following PSTK knockout (Fig. 5C). We observed the downregulation of several key ferroptosis-related protective factors in these PSTK-KO cells (Fig. 5D). Both Sorafenib and Erastin can induce ferroptosis, and our CRISPR screening results suggest that PSTK contributes to HCC cell resistance to Sorafenib or Erastin. Based on these results, we hypothesized that PSTK may protect HCC cells against the induction of ferroptosis.

### PSTK regulates GSH metabolism and folate biosynthesis

We next conducted a metabolomics study in order to examine the impact of PSTK knockout on metabolite levels in Hep3B cells. KEGG enrichment analyses of identified differentially abundant metabolites revealed them to be associated with oxidative phosphorylation (Fig. 5E), consistent with our RNA-seq results (Fig, 5A). Typically, oxidized phospholipids levels were dramatically increased following PSTK knockout, consistent with an increase in lipid peroxide levels associated with increased ferroptotic activity (Fig. 5G). Notably, the knockout of PSTK resulted in a dramatic decrease in GSH together with an increase in the levels of oxidized glutathione (GSSG). As key components of the GSH synthesis process [23], γ-glutamylcysteine, cysteinylglycine, and cysteine levels were significantly reduced, whereas no significant changes in cystine or glutamic acid levels were observed (Fig. 5G-H). This suggested that PSTK knockout specifically disrupted the process of cysteine synthesis. Moreover, significant reductions in the levels of key folate biosynthesis-related metabolites including folic acid and 5-Methyltetrahydrofolic acid were observed (Fig. 5H). A comprehensive analysis of both RNA-seq and metabolomics data indicated that the genes and metabolites associated with folate biosynthesis in these cells were highly correlated with one another (Fig. S6). These findings thus suggested that PSTK can protect HCC cells against the induction of ferroptosis by maintaining GSH metabolism and folate biosynthesis.

### PSTK contributes to HCC cell resistance to targeted therapy-induced ferroptosis

**Fig. 6:**
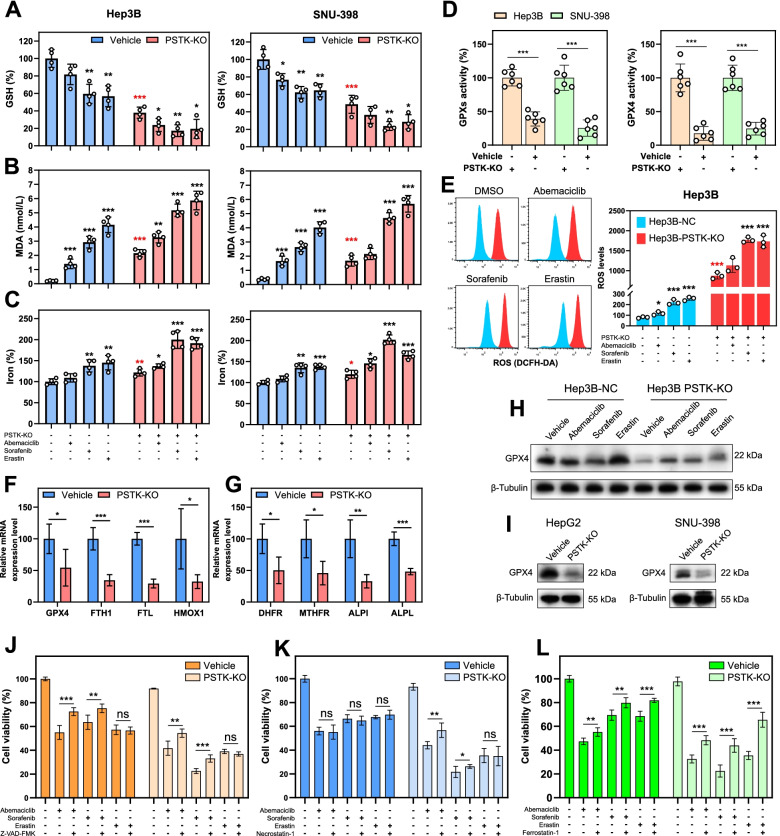
PSTK maintains GPX4 activity to protect against ferroptotic induction. **A-C** PSTK-knockout HCC cells were treated with Abemaciclib (5 μM), Sorafenib (5 μM), or Erastin (10 μM) for 24 h, after which levels of GSH, MDA, and iron were assessed (n=4). Comparisons were made between cells treated with inhibitors and vehicle-treated cells (black), or between PSTK-KO vs. PSTK-NC cells (red) **D** GPXs and GPX4 activities in PSTK-KO and control cells were assessed (n=6). **E** Knockout of PSTK promoted increases in ROS levels at baseline and in response to therapeutic treatment (n=3). Comparisons were made between cells treated with inhibitors and vehicle-treated cells (black), or between PSTK-KO vs. PSTK-NC cells (red). **F** GPX4, FTH1, FTL, and HMOX1 mRNA levels were measured via qPCR (n=4). **G** DHFR, MTHFR, ALPI, and ALPL mRNA levels were measured via qPCR (n=4). **H-I** Immunoblotting revealed significant GPX4 downregulation in PSTK-knockout HCC cells. **J-L** Hep3B-NC and Hep3B-PSTK-KO cells were treated with targeted therapies (Abemaciclib, 5 μM; Sorafenib, 5 μM; Erastin, 10 μM) together with or without inhibitors (Z-VAD-FMK, 10 μM; Necrostatin-1, 10 μM; Ferrostatin-1, 10 μM) for 48 h, and the inhibition of growth was assessed via CCK-8 assay (n=5). *p<0.05; **p<0.01; ***p<0.001; Student’s t-test

The selenium-containing enzyme glutathione peroxidase 4 (GPX4) is a central regulator of ferroptosis that can mitigate the toxicity of this caustic process [24]. PSTK, as a key kinase involved in the biosynthesis of selenocysteine, which forms the active center of GPX proteins, can thus contribute to resistance to targeted therapy-induced ferroptosis. In contrast to PSTK, GPX4 is overexpressed in most HCC cell lines and normal hepatocytes as compared to HUVECs (Fig. 2G). We found that PSTK-KO HCC cells exhibited increases in events consistent with ferroptosis, including the depletion of GSH, increased MDA production, and elevated iron levels (Fig. 6A-C). Abemaciclib, Sorafenib, or Erastin resulted in even more pronounced ferroptotic signaling in these cells (Fig. 6A-C). Consistently, PSTK-KO HCC cells exhibited lower baseline GPXs and GPX4 activities (Fig. 6D). PSTK-KO Hep3B cells also presented with higher baseline levels of ROS, and these levels were further amplified upon targeted therapy treatment (Fig. 6E). In subsequent qPCR assays, we found that PSTK was able to regulate the transcription of several key genes related to ferroptosis and folate biosynthesis (Fig. 6F-G). The knockout of PSTK resulted in significant decreases in GPX4 protein levels (Fig. 6H-I), in line with our RNA-seq and qPCR results. GPX4 was the only GPXs family member that was downregulated at the transcriptional level following PSTK knockout (Fig. 6F). Both direct and indirect targeting mechanisms, including GSH depletion, can lead to GPX4 inactivation. In addition to System Xc- inhibition, the direct inhibition of GSH synthesis can also induce ferroptosis [8]. Metabolomics analyses revealed that PSTK depletion generally impaired GSH synthesis by disrupting the conversion of cystine to cysteine (Fig. 5G-H). We hypothesized that knocking out PSTK was sufficient to impair selenocysteine synthesis, in turn deactivating other selenoproteins including thioredoxin reductase (TrxR), which plays a central role in the regulation of redox signaling [25] and contributes to the depletion of cysteine following PSTK knockout. We found that PSTK-KO HCC cells have exhibited both reduced cysteine concentrations and lower TrxR activities (Fig. S7), consistent with CRISPR screening results that TXNRD1/TXNRD3 were associated with Sorafenib/Erastin treatment resistance in Hep3B cells (Fig. 2A). As such, PSTK is able to protect HCC cells against the induction of ferroptotic cell death at least in part by maintaining GPX4 activity and GSH synthesis. To analyze other types of alternative cell death pathways triggered by chemotherapy in PSTK knockout cells, Z-VAD-FMK (apoptosis inhibitor), Necrostatin-1 (necrosis inhibitor) or Ferrostatin-1 (ferroptosis inhibitor) were used to attempt to rescue potential cell death. As shown in Fig. 6J-L, Z-VAD-FMK was able to moderately inhibit Abemaciclib/Sorafenib-induced cell death in PSTK-NC/PSTK-KO cells and failed to inhibit Erastin-induced cell death (Fig. 6J). Necrostatin-1 could only slightly inhibit Abemaciclib/Sorafenib-induced cell death in PSTK -KO cells (Fig. 6K). However, Ferrostatin-1 significantly inhibited Abemaciclib, Sorafenib and Erastin induced cell death in PSTK-KO cells. These results indicated that a mixed form of ferroptotic and apoptotic cell death occurs in PSTK-KO cells under Abemaciclib/Sorafenib treatment (Fig. 6L). As such, we hypothesized that ferroptosis play a major role in PSTK-regulated cell death under treatment with typical ferroptosis inducers (Sorafenib and Erastin).

### Punicalin/Geraniin are PSTK inhibitors that exhibit synergistic efficacy when employed together with Sorafenib

**Fig. 7:**
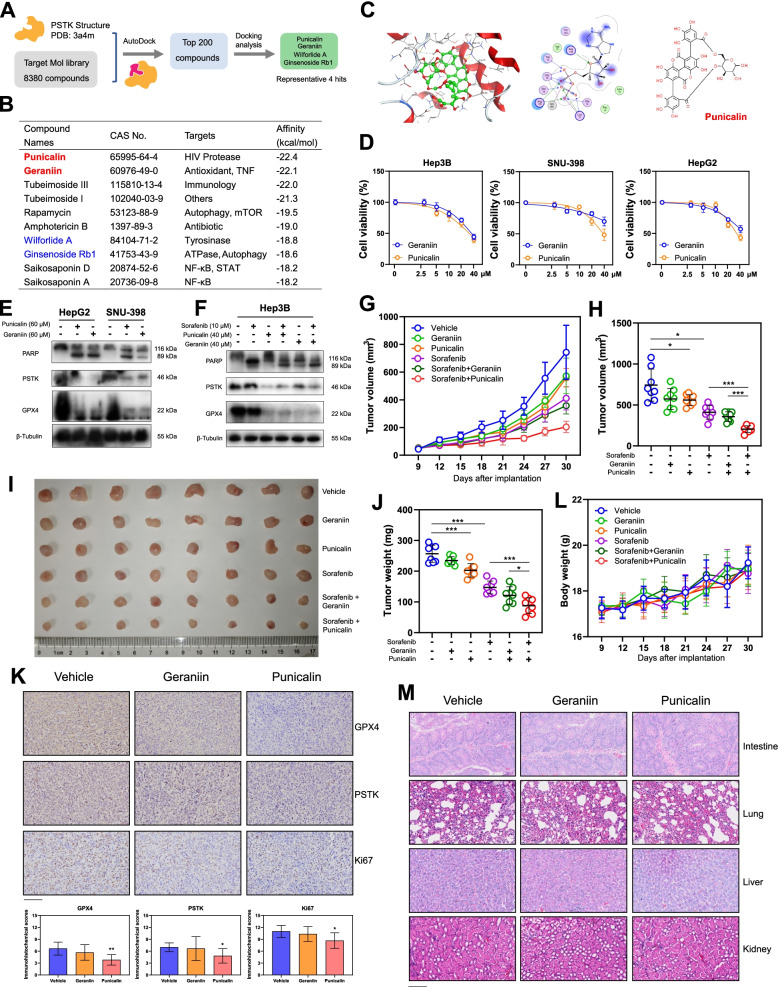
Punicalin is a potential PSTK inhibitor that synergizes with Sorafenib *in vitro* and *in vivo*. **A** Schematic overview of the high throughout virtual screening approach used for PSTK inhibitor identification. **B** Docking scores for the top 10 potential PSTK inhibitors. **C** Punicalin binds to the active pocket of PSTK through six H-bonds formed with Lys200, Asp41, Asp79, Thr80, Tyr82, and Met86. **D** Geraniin and Punicalin exhibited significant anti-HCC activities by CCK-8 assay (n=6). **E-F **Punicalin/Geraniin treatment for 48 h significantly downregulates PSTK and GPX4 at the protein level. **G** Changes in tumor volumes over time for mice implanted with Hep3B cells and treated with Sorafenib with or without Punicalin/Geraniin. **H-J** Measures of tumor volume and tumor weight values of mice at the study endpoint. **K** Representative images (six random visual fields) of GPX4, PSTK and Ki-67 staining in tumor samples from the Punicalin/Geraniin/Vehicle treatment groups and immunohistochemical scores (n=6). Scale bar: 100 μm. **L** Changes in murine body weights over time. **M** Representative images (six random visual fields) of H&E staining of intestine, lung, liver, and kidney samples from Punicalin/Geraniin/Vehicle treated mice. Scale bar: 100 μm. *p<0.05; **p<0.01; ***p<0.001; Student’s t-test

**Fig. 8:**
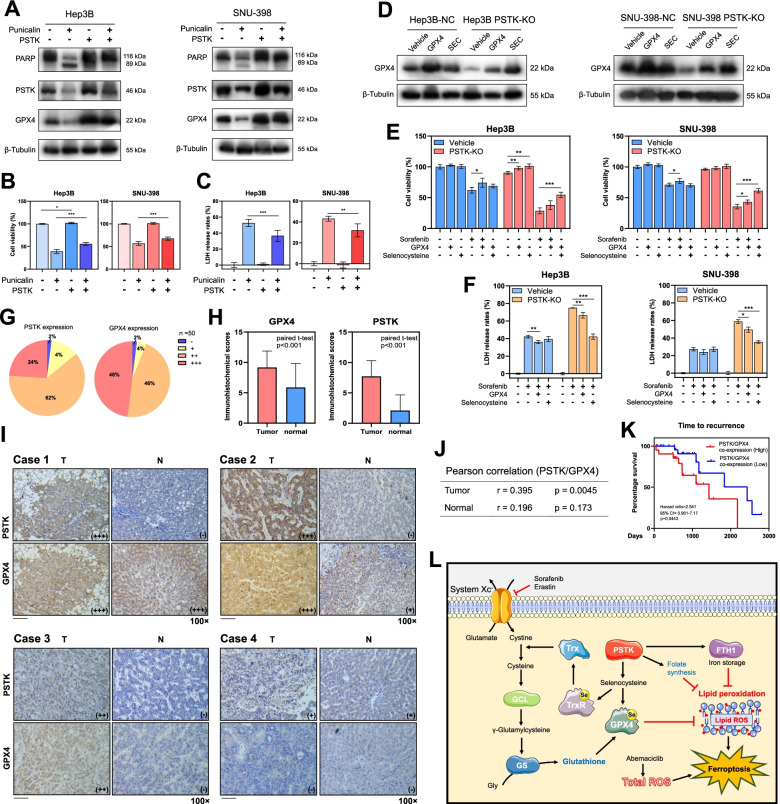
HCC patients exhibit PSTK overexpression that is correlated with the expression of GPX4. **A** Western blot showed the effect of recovering PSTK/GPX4 expressions with PSTK overexpressing for 48 h on Punicalin-induced PSTK/GPX4 deficiency and cleavage of PARP. **B-C** CCK-8 assays (n=6) and LDH release assays (n=6) showed the rescue effect with PSTK overexpressing for 48 h on Punicalin induced cell death. **D** Western blotting revealed the recovery of GPX4 expression following GPX4 overexpressing or selenocysteine treatment (SEC, 5 μM) for 48 h in the context of PSTK-KO induced GPX4 deficiency. **E-F** CCK-8 assays (n=4) and LDH release assays (n=3) demonstrated the rescue effects associated with GPX4 overexpression or selenocysteine treatment for 48 h on PSTK-KO induced cell death under sorafenib treatment. *p<0.05; **p<0.01; ***p<0.001; Student’s t-test. **G** PSTK expression in 50 pathologically confirmed HCC tissues and paired normal liver tissue samples as assessed via immunohistochemical staining, with expression being scored as high (+++), intermediate (++), low (+), or negative (-). **H** PSTK/GPX4 expression in 50 HCC tissues and paired non-tumor tissues (Paired t-test, p<0.0001). **I** Typical immunohistochemical staining results for PSTK/GPX4 staining in HCC tissues and paired non-tumor tissues. Scale bar: 100 μm. **J** Pearson correlation analyses of the relationship between PSTK/GPX4 immunohistochemical scores in 50 HCC samples or normal tissue samples. **K** Kaplan-Meier curves demonstrating the relationship between high levels of PSTK/GPX4 co-expression and shorter time to recurrence following surgery. **L** Schematic overview of the proposed mechanism whereby PSTK protects HCC cells against ferroptosis

To further evaluate the potential clinical feasibility of targeting PSTK as a means of treating HCC, we next conducted a structure-based virtual screen of 8380 compounds using TargetMol in an effort to identify PSTK inhibitors. The docking scores for the top 10 compounds are shown in Fig. 7B. Based upon binding mode analyses, four representative compounds were selected, and associated 2D/3D figures were generated as shown in Fig. 7C and Fig. S8A. Interestingly, two hydrolyzable tannins (Punicalin and Geraniin) exhibited the highest binding affinity for the active pocket of PSTK, and both have been identified as novel anti-HBV agents in prior research [26]. Both Punicalin and Geraniin exhibited moderate cytotoxic efficacy when used to directly treat HCC cells (Fig. 7DD and S8B), but significantly sensitized HCC cells to Sorafenib (Fig. S8C-D). Consistent with PSTK knockout, Punicalin or Geraniin treatment induced significant decreases in GPX4 protein levels in these tumor cells (Fig. 7E-F). To assess the clinical relevance of these results, we conducted an animal study in which Punicalin/Geraniin were orally administered together with Sorafenib. We found that Punicalin treatment significantly inhibited tumor growth and synergized with Sorafenib treatment (Fig. 7G-J). Immunohistochemical staining revealed that Punicalin treatment significantly downregulated GPX4, PSTK and Ki-67 activities in tumor samples (Fig. 7K). Both Geraniin and Punicalin were well-tolerated with no significant weight loss in mice in the monotherapy and combination treatment groups (Fig. 7L). H&E staining demonstrated that there were no significant morphological changes in major organs of mice (Fig. 7M). As such, disrupting PSTK kinase activity using Punicalin may represent an effective means of overcoming Sorafenib resistance in HCC. To validate the specificity of Punicalin for targeting PSTK, we performed rescue experiments by overexpressing PSTK and observed that protein levels of GPX4 and PSTK significantly recovered after PSTK overexpression under Punicalin treatment (Fig. 8A). PSTK overexpression also rescued HCC cells from Punicalin-induced cell death (Fig. 8B-C)

### PSTK regulates GPX4 expression in a selenocysteine dependent manner

To further analyze how PSTK regulates GPX4 activity, we performed rescue experiments by treating PSTK-KO cells with a GPX4 overexpression plasmid or selenocysteine (5 μM). We found that GPX4 overexpression was able to partially recover GPX4 and slightly rescued PSTK-KO cells from Sorafenib-induced cell death (Fig. 8D-F). However, the rescue effects of selenocysteine treatment were stronger than those observed following GPX4 overexpression (Fig. 8D-F), indicating a selenocysteine-dependent regulatory mechanism.

### PSTK expression is correlated with GPX4 levels in HCC samples

To explore the expression of PSTK in HCC patients, we next utilized an immunohistochemistry auto-Stainer approach to evaluate PSTK expression in a 209-nodule tissue microarray (Fig. S9), which included 169 pathologically confirmed HCC tissues and 34 hepatobiliary adenocarcinoma tissues. PSTK positivity was evident in 108 of these HCC samples (Fig. S9). Associations between PSTK and GPX4 expression were next assessed in 50 pairs of HCC patient tumor tissues and paired paracancerous liver tissue samples, revealing the marked upregulation of both of these genes in HCC samples relative to matched control tissues (Fig. 8G-I), consistent with mRNA levels detected in the TCGA database (Fig. 2B). Pearson correlation analyses indicated PSTK and GPX4 expression were significantly correlated in HCC samples (r=0.395, p=0.0045), while no such correlation was evident in normal liver samples (Fig. 8J). In univariate analyses, both cirrhosis and GPC3 were identified as risk factors associated with HCC recurrence, whereas PSTK/GPX4 expression did not predict the prognosis of these HCC patients (Table S2). PSTK/GPX4 co-expression values were calculated by multiplying together the immunohistochemical scores for these two targets and then grouping these 50 HCC patient samples into low and high co-expression groups based on whether they were above or below the median co-expression value. We found that the time to recurrence was shorter for patients with higher levels of PSTK/GPX4 co-expression in this cohort (Fig. 8K). As such, these data suggested that PSTK and GPX4 may serve as valuable prognostic biomarkers in HCC patients.

## Discussion

Since its initial discovery, a growing body of evidence has demonstrated the potential value of targeting ferroptosis as a means of treating drug-resistant tumors. The determination that first-line Sorafenib treatment can induce ferroptosis has further supported the promise of ferroptosis-inducing therapeutic agents. Indeed, researchers have found that triggering ferroptosis by inhibiting the mechanisms that normally reduce the cytotoxicity of this mechanism may represent an effective means of overcoming targeted therapy resistance in HCC [6, 9, 27]. However, much work remains to be done to clarify the mechanisms underlying ferroptosis in the context of oncogenesis. GPX4 is a key detoxifying protein associated with ferroptosis which functions as a lipid peroxide scavenger [24]. When GPX4 is inactivated via direct or indirect targeting, this can result in the induction of ferroptotic cancer cell death [6, 24]. GPX4 is a selenium-dependent enzyme with a selenocysteine (Sec)-containing active site, and ferroptosis inducers that covalently bind to this Sec site can directly suppress GPX4 activity [8]. Early work suggests that decreases in selenium levels can drive increased lipid peroxide accumulation in HCC cells [28]. The selenocysteine biosynthesis pathway is required for the survival of cancer cells [29]. As such, enzymes that target selenocysteine biosynthesis have the potential to suppress GPX4 activity and to enhance the induction of ferroptosis in HCC cells.

Through a series of CRISPR/Cas9 knockout library screens together with RNA-seq and metabolomics analyses, we identified PSTK, which is a pivotal intermediate involved in selenocysteine biosynthesis [20], as a key mediator of HCC cell targeted therapy resistance. In RNA-seq analyses, PSTK was found to be closely linked to the regulation of ferroptosis, and the knockout of PSTK resulted in the upregulation of ferroptosis-related genes in HCC cells. Importantly, we found that while PSTK knockout alone did not directly induce ferroptotic cell death, it did markedly increase the sensitivity of HCC cells to subsequent targeted therapy treatment. PSTK knockout also resulted in a significant decrease in GPX4 expression and activation in a selenocysteine dependent manner, suggesting that the regulation of GPX4 may be a primary mechanism whereby this protein protects against ferroptotic induction. Ferroptosis has been considered as a double-edged sword in the treatment of cancer, and the mechanisms of ferroptosis regulation in HCC are particularly important [6, 29]. We found that PSTK was significantly upregulated in HCC tumor tissue samples as compared to normal liver tissues in the TCGA database and in a cohort of HCC patient samples from our center. GPX4 protein levels were correlated with PSTK protein levels in HCC tissue samples, whereas no such correlation was evident in control liver tissues. As such, we speculate that PSTK may represent an HCC-specific therapeutic target associated with minimal cytotoxicity in normal cells.

In addition to GPX4 inhibition, intracellular GSH depletion is another key driver of ferroptosis induction [6]. GSH functions as a co-factor that enables GPX4 to eliminate lipid peroxides from cells. The synthesis of GSH is dependent upon System Xc- through the exchange of extracellular cystine for intracellular glutamate [10]. Cystine is reduced to yield cysteine, which is the most important intermediate metabolite involved in GSH synthesis [30]. In certain cancers, cysteine depletion has been shown to induce ferroptosis and thus represents an attractive therapeutic approach [31]. We demonstrated that PSTK was able to sustain GSH synthesis via maintaining the reduction of cystine to cysteine. Such PSTK-dependent cysteine synthesis may be carried out in part by the TrxR/Trx system, which is composed of selenocysteine-dependent antioxidant enzymes [32, 33]. Notably, we also found that PSTK depletion resulted in marked reductions in folate biosynthesis. Our comprehensive RNA-seq and metabolomics analyses revealed that mRNA levels were significantly correlated with levels of metabolites involved in the process of folate biosynthesis, suggesting that PSTK may regulate this metabolic process at the transcriptional level. Recent work has demonstrated that tetrahydrobiopterin (BH4) availability plays a key protective role in the control of cellular responses to ferroptosis upon GPX4 inhibition [34]. As such, we hypothesize that PSTK may protect HCC cells against the induction of ferroptosis in a GPX4-independent manner by regulating folate synthesis at the transcriptional level. Our results also suggest that PSTK may promote the upregulation of FTH1 and FTL to further protect HCC cells against ferroptotic induction (Fig. 8L).

Our analyses suggested that PSTK represents a promising target to overcome HCC cell resistance to targeted therapy. Through a virtual screening approach, we identified the hydrolyzable tannins Punicalin and Geraniin as potential PSTK inhibitors. Punicalin increased sensitivity of HCC cells to Sorafenib treatment and was associated with GPX4 downregulation, consistent with the results of PSTK knockout in our CRISPR screen. Punicalin notably functions by inhibiting HBV covalently closed circularDNA (cccDNA) production and promote cccDNA decay [26]. Given that HBV remains the most important global risk factor associated with HCC incidence, we posit that combined Punicalin and Sorafenib treatment may represent a promising approach to HCC patient therapy, particularly for patients with HBV-associated HCC.

## Conclusions

In summary, our study is the first to have identified PSTK as a critical mediator of ferroptosis resistance in HCC cells. Mechanistically, PSTK depletion was associated with the suppression of selenocysteine-dependent GPX4 activation, GSH metabolism, and folate synthesis, resulting in the induction of sublethal ferroptosis that rendered HCC cells more sensitive to targeted therapies capable of inducing ferroptosis. As such, inhibiting PSTK may represent a novel and viable approach to overcoming targeted therapy resistance in HCC patients, and inhibitors of this protein thus warrant further clinical evaluation.

## Supplementary Information

**Additional file 1.**

## Data Availability

All data supporting this study and its findings are available within the article. Datasets of sgRNA sequencing and RNA-seq data are deposited in the GEO database (GSE182185).

## Abbreviations

CRISPRf: Clustered regularly interspaced short palindromic repeats
HCC: Hepatocellular carcinoma
PSTK: Phosphoseryl-tRNA kinase
GPX4: Glutathione peroxidative 4
GSH: Glutathione
GSSG: Oxidized glutathione
HBV: Hepatitis B virus
TKI: Tyrosine kinase inhibitor
CDK: Cyclin-dependent kinases
OS: Overall survival
ORR: Objective response rate
sgRNA: Small guide RNA
KO: Knockout
KEGG: Kyoto Encyclopedia of Genes and Genomes
SOD2: Manganese Superoxide Dismutase
TCGA: The cancer genome atlas
MDA: Malondialdehyde
TrxR: Thioredoxin reductase
RNA-seq: RNA sequencing
BH4: Tetrahydrobiopterin
LDH: Lactate dehydrogenase
SEC: Selenocysteine
H&E: Hematoxylin and eosin
