# Supplementary material for: CRISPR screens uncover protective effect of PSTK as a regulator of chemotherapy-induced ferroptosis in hepatocellular carcinoma
Source: Mol Cancer. 2022 Jan 4;21:11. doi: 10.1186/s12943-021-01466-9 (PMC8725338; doi:10.1186/s12943-021-01466-9)
Supplement: Supplementary file 1 — Additional file 1. [file 12943_2021_1466_MOESM1_ESM.docx]

**CRISPR screens uncover protective effect of PSTK as a regulator of chemotherapy-induced ferroptosis in hepatocellular carcinoma**

Yiran Chen, Li Li, Jie Lan, Yang Cui, Xiaosong Rao, Jing Zhao, Tao Xing, Gaoda Ju, Guangtao Song, Jizhong Lou, Jun Liang

Table of contents-------------------------------------------------------------------------------1

Supplementary materials and methods --------------------------------------------------2

Supplementary Figures -------------------------------------------------------------------- 11

Supplementary Tables --------------------------------------------------------------------- 21

References ----------------------------------------------------------------------------------- 24

**Supplementary material and methods**

**Cell lines**

Human HCC cell lines (Hep3B, HepG2, SK-HEP-1, PLC/PRF/5, SNU-387, SNU-182, SNU-398), human normal hepatocyte (WRL68) and human umbilical vein endothelial cells (HUVECs) were purchased from American Type Culture Collection (ATCC). HCC cell lines (JHH2, JHH7 and Huh7) were purchased from Japanese Collection of Research Bioresources Cell Bank (JCRB). HCC cell line Li-7 was purchased from Procell Life Science (Wuhan, China). All cell lines were authenticated by STR profile. All cells were cultured in Dulbecco’s modified eagle medium (DMEM, Gibco) with 1% penicillin/streptomycin (Gibco) and 10% fetal bovine serum (FBS, Gibco) at 37 °C / 5% CO_2_. 293FT cells were cultured without penicillin/streptomycin.

**Antibodies and inhibitors**

Antibodies against GPX4 (67763-1-Ig, WB 1:2000, IHC 1:2000) was purchased from Proteintech. PARP (#9542, 1:1000) and β-Tubulin (#2146, 1:5000) were purchased from Cell Signaling Technology. Antibodies against PSTK (ab272622, IHC 1:100) were purchased from Abacam. Antibody against PSTK (sc-373991, WB 1:100) was purchased from Santa Cruz Biotechnology. Abemaciclib (S5716), Palbociclib (S1579), Sorafenib (S7397), Lenvatinib (S1164), Regorafenib (S1178), Erastin (S7242), Z-VAD-FMK (S7023), Necrostatin-1 (S8037) and Ferrostatin-1 (S7243) were purchased from Selleck Chemicals. Punicalin (HY-N0639) and Geraniin (HY-N0472) were purchased from MCE. Selenocysteine (10236-58-5) were purchased from TOPULE (Wuhan, China).

**In vitro cell viability/death analyzed by CCK-8 assay, LDH release assay and colony formation**

For short term assays, 5000 cells were seeded at 96-well pates and cultured overnight. Then cells were treated with inhibitors (Abemaciclib, Sorafenib, Erastin or vehicle) for hours and cell viability/death were analyzed by CCK-8 (DOJINDO) assay and LDH release assay (DOJINDO). For long term assays, 800 cells were seeded at 6-well plates in triplicate and cultured overnight. Then cells were treated with inhibitors or vehicle for 10 days and stained with crystal violet dye (Sigma-Aldrich). Colony numbers were measured by ImageJ software (1.48v).

**Protein preparation and western blotting**

The total protein lysates were extracted by RIPA buffer (KeyGEN Biotech) and the protein concentrations were determined using BCA Protein Assay Kit (ZJ101, EpiZyme). Samples were separated by SDS-PAGE electrophoresis and transferred to PVDF membrane. Protein bands were incubated with indicated antibodies and determined using Omni-ECL™Femto Light Chemiluminescence Kit (SQ201, EpiZyme).

**Real-time PCR**

Total RNA isolation from cells was conducted according to the manufacturer's instructions for TRIzol reagent (TAKARA). RNA was reverse transcribed into cDNA using a PrimeScript RT reagent kit (TAKARA). RT-PCR analysis was performed using a SYBR Green Dye detection system (Applied Biosystems). The transcript levels were expressed as fold change compared to vehicle samples. The primers for RT-PCR were listed in **Table S3**.

**Apoptosis, cell cycle and ROS measurement by flow cytometry**

Apoptosis was measured using the Annexin V/7-AAD Apoptosis Detection Kit according to the manufacturer’s protocol (BioLegend). Cell cycle was determined by measuring DNA content using propidium iodide (PI) staining. Cells were seeded at 6-well plates for triplicate and cultured for appropriate time until the cells at 70% confluence. For whole cell ROS measurement, cells were cultured with fresh DMEM with inhibitors or vehicle for 24 hours and stained with 10 μM 2',7'-Dichlorodihydrofluorescein diacetate (DCFH-DA) (KeyGEN BioTECH). Apoptosis, cell cycle and ROS level were assayed by flow cytometry and analyzed by FlowJo software (V10).

**Measurement of cellular ferroptosis levels**

The glutathione (GSH) concentrations were analyzed using a Glutathione Assay Kit (#BC1175, Solarbio) according to manufacturer’s instructions. The malondialdehyde (MDA) concentrations were analyzed using a Lipid Peroxidation (MDA) Assay Kit (#BC0025, Solarbio) according to manufacturer’s instructions. The iron concentrations were analyzed using an Iron Assay Kit (#BC4355, Solarbio) according to manufacturer’s instructions. The relative glutathione peroxidase (GPXs) activities were analyzed using a GPXs Assay Kit (#BC1190, Solarbio) according to manufacturer’s instructions. The relative glutathione peroxidase 4 (GPX4) activities were analyzed using a GPX4 ELISA kit (#RF5693, Ruifan Biotechnology). The relative thioredoxin reductase (TrxR) activities were analyzed using a TrxRs Assay Kit (#BC1155, Solarbio) according to manufacturer’s instructions. The cysteine concentrations were analyzed using a Cysteine (Cys) Assay Kit (#BC0185, Solarbio) according to manufacturer’s instructions.

**RNA sequencing**

For RNA sequencing, RNA integrity was assessed using the RNA Nano 6000 Assay Kit of the Bioanalyzer 2100 system (Agilent Technologies, CA, USA). Differential expression analysis of two groups (PSTK-KO vs. vehicle, three biological replicates) was performed using the DESeq2 R package (1.20.0). Genes with an adjusted P-value (Padj) <0.05 found by DESeq2 were assigned as differentially expressed. We used clusterProfiler R package to test the statistical enrichment of differential expression genes in KEGG pathways. Gene set enrichment analysis (GSEA) was performed using GSEA software (4.1.0).

**Untargeted metabolomics**

Metabolites from samples (Three biological replicates) were extracted by sonification and dissolved with 10% methanol. The solution was injected into the LC-MS/MS system analysis^1,2^. The raw data files generated were processed using the Compound Discoverer 3.1 (CD3.1, ThermoFisher) to perform peak alignment, peak picking, and quantitation for each metabolite. And then peaks were matched with the mzCloud (https://www.mzcloud.org/)，mzVault and MassList database to obtain the accurate qualitative and relative quantitative results. These metabolites were annotated using the KEGG, HMDB and LIPIDMaps. Principal components analysis (PCA) and Partial least squares discriminant analysis (PLS-DA) were performed at metaX^3^. The metabolites with VIP > 1 and P-value< 0.05 and fold change≥2 or FC≤0.5 were considered to be differential metabolites. Volcano plots were used to filter metabolites of interest which based on log2(FoldChange) and -log10(p-value) of metabolites.

**Generation of HCC spheroids**

To generate spheroids, PSTK-KO or vehicle cells suspended in complete DMEM medium were seeded at a density of 2000 cells/well in 96-well round-bottomed ultra-low attachment surface (Corning). The plates were incubated for 21 days and taken a picture every day. Spheroid volumes were measured by modified ellipsoidal formula, spheroid volume = (length × width^2^)/2.

**Immunohistochemical staining of HCC samples**

A tissue microarray containing 169 nodules of HCC patients were purchased from Avilabio (Shanxi, China). Immunohistochemical staining for PSTK (1:100) was done using Immuno-Histo Stainer. We collected HCC specimens and paired normal liver specimens from 50 patients who underwent surgery in Peking University International Hospital of the Peking University in Beijing, China. All patients did not receive any treatment before surgery. Immunohistochemical staining for PSTK (1:100) and GPX4 (1:2000) were done. Two pathologists independently assessed immunohistochemical score. The immunostaining score was evaluated on the basis of percentage score × intensity score. The correlations of immunostaining scores between PSTK and GPX4 were analyzed by Pearson correlation analysis. Clinical characteristics and PSTK/GPX4 expression were compared by chi-square test. The cumulative recurrent time was estimated using the Kaplan-Meier method. Univariate analyses were performed using the Cox proportional hazards model.

**High-throughout virtual screen for potential PSTK inhibitors**

AutoDock Vina^4^, an open-source program for molecular docking, was used for structure-based VS (SBVS). The X-ray structure of PSTK (UniProtKB - Q58933) was downloaded from RCSB Protein Data Bank (PDB ID: 3a4m) and the chain A was defined as receptor. The data from TargetMol were used as VS library, which totally includes 8380 compounds. Figure 1 shows the flowchart of SBVS. All compounds were prepared with the Wash module in MOE 2019.0102. After that, all compounds had been transformed into pdbqt files by openbabel. The receptor was prepared with AutoDockTools. The parameter of SBVS can be found in the conf.txt file. The molecules had been ranked by the affinity value in the high throughput rigid docking results. After SBVS, the information of ranked top 200 compounds from the library was saved. The docking modes of 4 representative hits have been analyzed via MOE. The 2D and 3D figures had been produced.

**Combination analyses of Sorafenib and PSTK inhibitors in vitro**

A 5 × 6 matrix was designed that contained four Sorafenib concentrations and five Geraniin/Punicalin concentrations for 48-hour treatments. All cells were seeded into 96 well plates and proliferated into 70% confluence before treatment. Then the percentages of cell death were measured by CCK-8 assay. The Chou-Talalay^5^ method and CompuSyn (V1.0) were used to quantify the effects, and the combination index (CI) values were calculated for each combination.

**Rescue experiments by overexpressing GPX4/PSTK**

GPX4 expression plasmid (ssAAV.CAG.mGPX4-P2A-EBFP.WPRE.SV40pA) and PSTK expression plasmid (ssAAV.CAG.mPSTK-P2A-EBFP.WPRE.SV40pA) were constructed by PackGene Biotech (Guangzhou, China). Hep3B-NC and Hep3B-PSTK-KO cells were transfected with GPX4 expression plasmid or selenocysteine, then detected protein levels of GPX4 and cell viabilities/death under Sorafenib treatment. Hep3B cells were transfected with PSTK expression plasmid, then detected protein levels of PSTK, GPX4 and PARP. Cell viabilities/death were detected after Punicalin treatment.

**Xenograft**

Animal experiments were carried out in accordance with Institute of Biophysics, Chinese Academy of Science’s Policy on Care and Use of Laboratory Animal. Female Nod-SCID mice of 6-8 weeks old were purchased from HFK BIOSCIENCE (Beijing). Hep3B-vehicle/Hep3B-PSTK-KO cells were harvested and injected subcutaneously (1 × 10^7^ cells in 200 μL PBS) into Nod-SCID mice (upper flank). Treatments were started when tumor volumes reached around 50 mm^3^. Included mice were randomly divided into four groups and injected intraperitoneally with Abemaciclib (50 mg/kg, every other day) or vehicle. Mice were sacrificed when the tumor volume exceeded 2000 mm^3^. PSTK-KO or vehicle Hep3B cells were implanted and treated with Sorafenib (50 mg/kg, every other day) or Erastin (50 mg/kg, every other day) for 42 days. Tumor volumes were monitored and quantified by the modified ellipsoidal formula, tumor volume = (length × width^2^)/2. To check the efficacities and appraisal the side effects of PSTK inhibitors, Hep3B cells were harvested and in injected subcutaneously (5 × 10^6^ cells in 200 μL PBS) into Nod-SCID mice (upper flank). Treatments were started when tumor volumes reached around 50 mm^3^. Included mice were randomly divided into six groups and intragastrically treated with Punicalin (100 mg/kg, every day), Geraniin (100 mg/kg, every day), Sorafenib (50 mg/kg, every day) with or without PSTK inhibitors (Punicalin/Geraniin) for 30 days. Tumor volumes and mice weights were measured every three days. Mice were sacrificed when the tumor volume exceeded 1000 mm^3^. Tumor samples from vehicle, Punicalin and Geraniin treated groups were excised, fixed, and embedded in paraffin for immunohistochemical analyses, protein expressions of GPX4, PSTK and Ki-67 were analyzed using immunohistochemical scores. Major organs of mice under vehicle/Punicalin/Geraniin treated were excised, fixed, and embedded in paraffin for H&E staining.

**Statistical analysis**

Data were shown as the mean ± standard deviation (SD). Paired or non-paired Student’s t test was used to compare the means of two groups using GraphPad Prism 8. The correlation analysis was performed using Pearson correlation method. Kaplan-Meier survival analysis was used to compare HCC patient survival using a log-rank test (GraphPad Prism 8). Clinical characteristics and PSTK/GPX4 expression were compared by chi-square test. Cox proportional hazards regression was used to analyze the effect of clinical variables on patient recurrence. P < 0.05 was considered to be statistically significant for all tests.

**Supplementary figures**


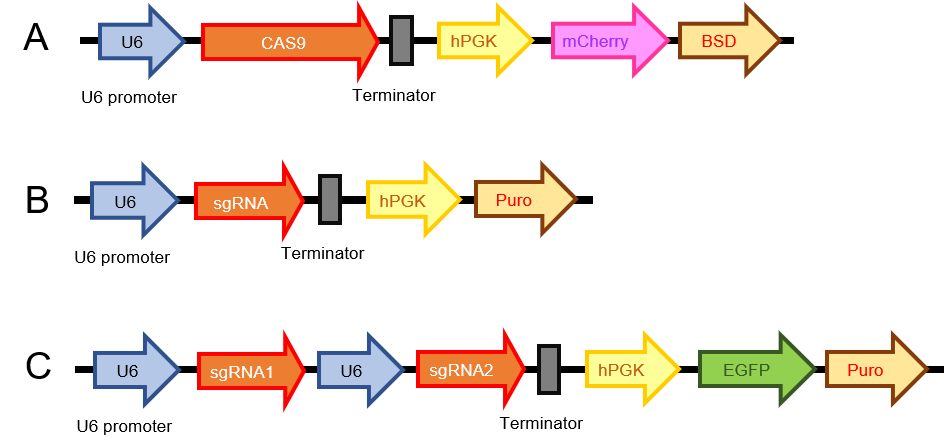


**Fig. S1. Schematic representation of lentiviral vectors. A.** Cas9-expressing vector. **B.** sgRNA-expressing vectors in TKOv3 library. **C.** Dual sgRNA-expressing vector for PSTK knockout. BSD, blasticidin selection gene; Puro, puromycin selection gene; hPGK，hPGK promoter; EGFP and mCherry, fluorescent tags.


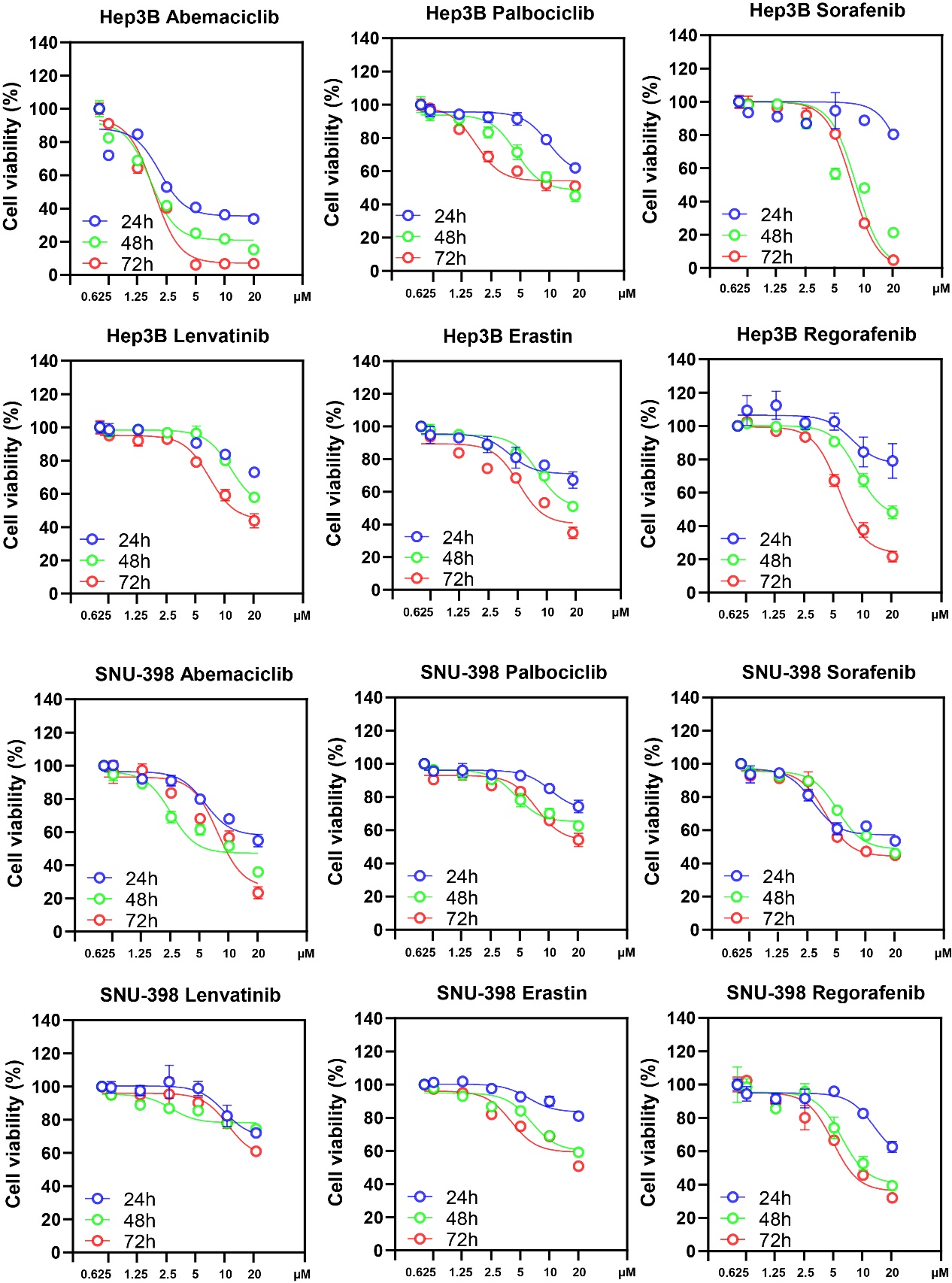


**Fig. S2. Cell viabilities of HCC cells after different therapies.** Hep3B and SNU-398 cells were treated with increasing concentrations of Abemaciclib, Palbociclib, Sorafenib, Lenvatinib, Regorafenib and Erastin for 24-72 hours. Cell viabilities were analyzed by CCK-8 assay.


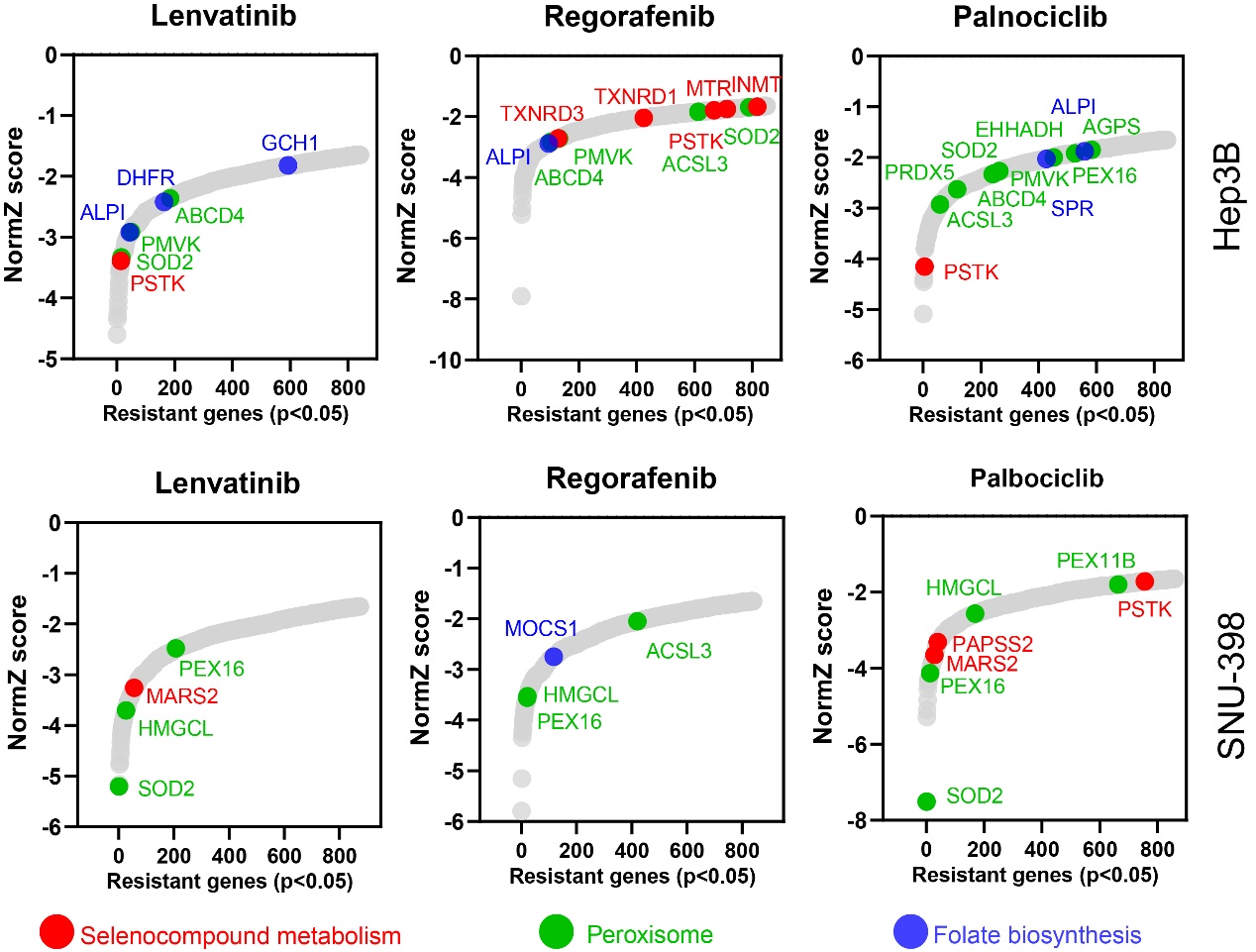


**Fig. S3. Genes from selenocompound metabolism, peroxisome and folate biosynthesis were strongly deleted in Lenvatinib, Regorafenib or Palbociclib treatment groups.** Typical genes from selenocompound metabolism, peroxisome and folate biosynthesis were marked by color dots.


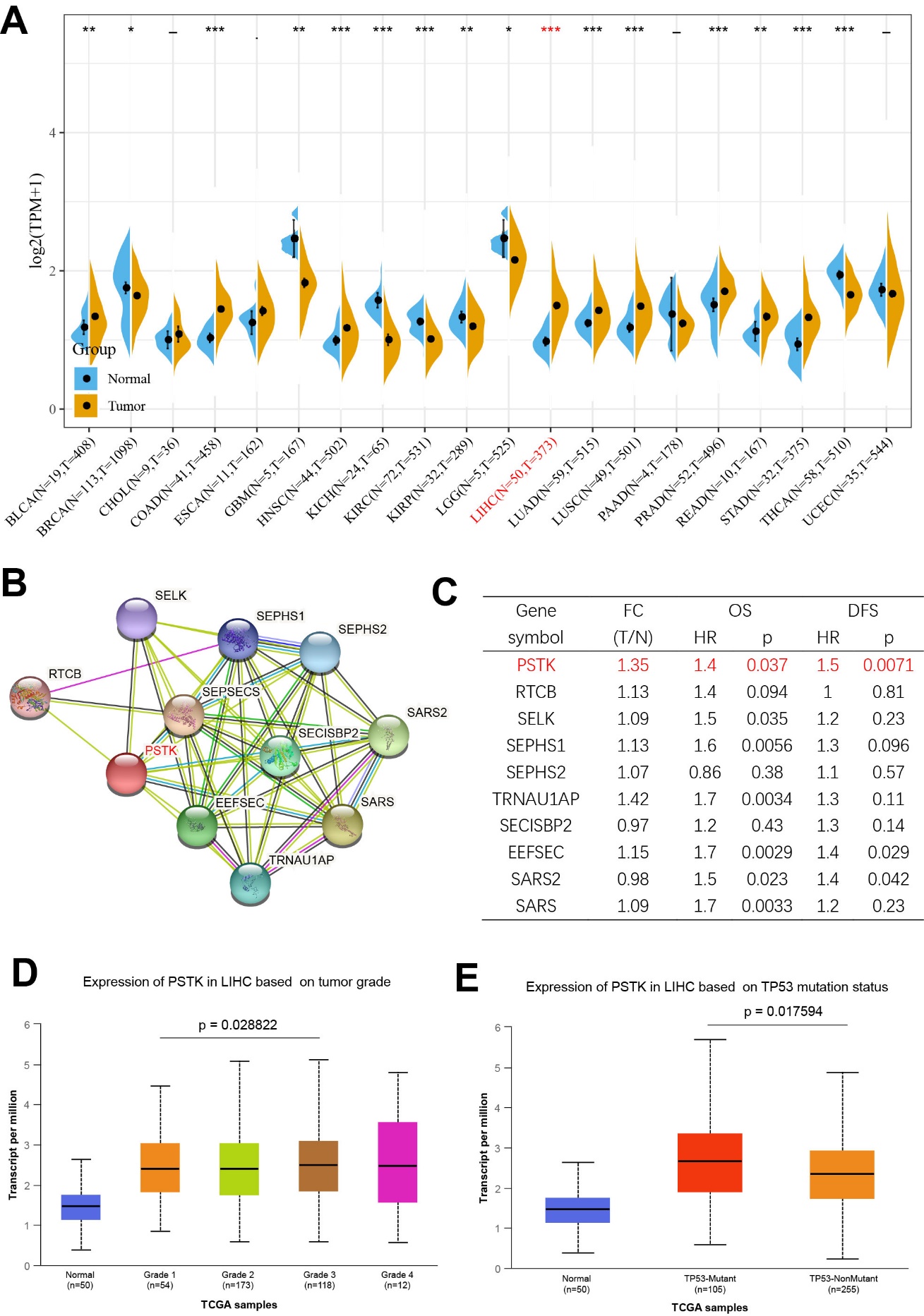


**Fig. S4. PSTK involved selenocysteine biosynthesis pathway is related to outcome of HCC patients. A.** The PSTK expression profile across all tumor samples and paired normal tissues from TCGA cohort. Student’s t test was used **B.** Functional protein association network (STRING) suggested PSTK was a relevant gene of selenocysteine biosynthesis. **C.** PSTK and selenocysteine biosynthesis related genes were overexpressed in HCC sample and higher gene expression exhibited worse prognosis. FC: foldchange, T/N: tumor/normal, OS: overall survival, DFS: disease free survival, HR: Hazard Ratio. **D.** Expression of PSTK in LIHC based on tumor grade (TCGA). **E.** Expression of PSTK in LIHC based on TP53 mutation status.


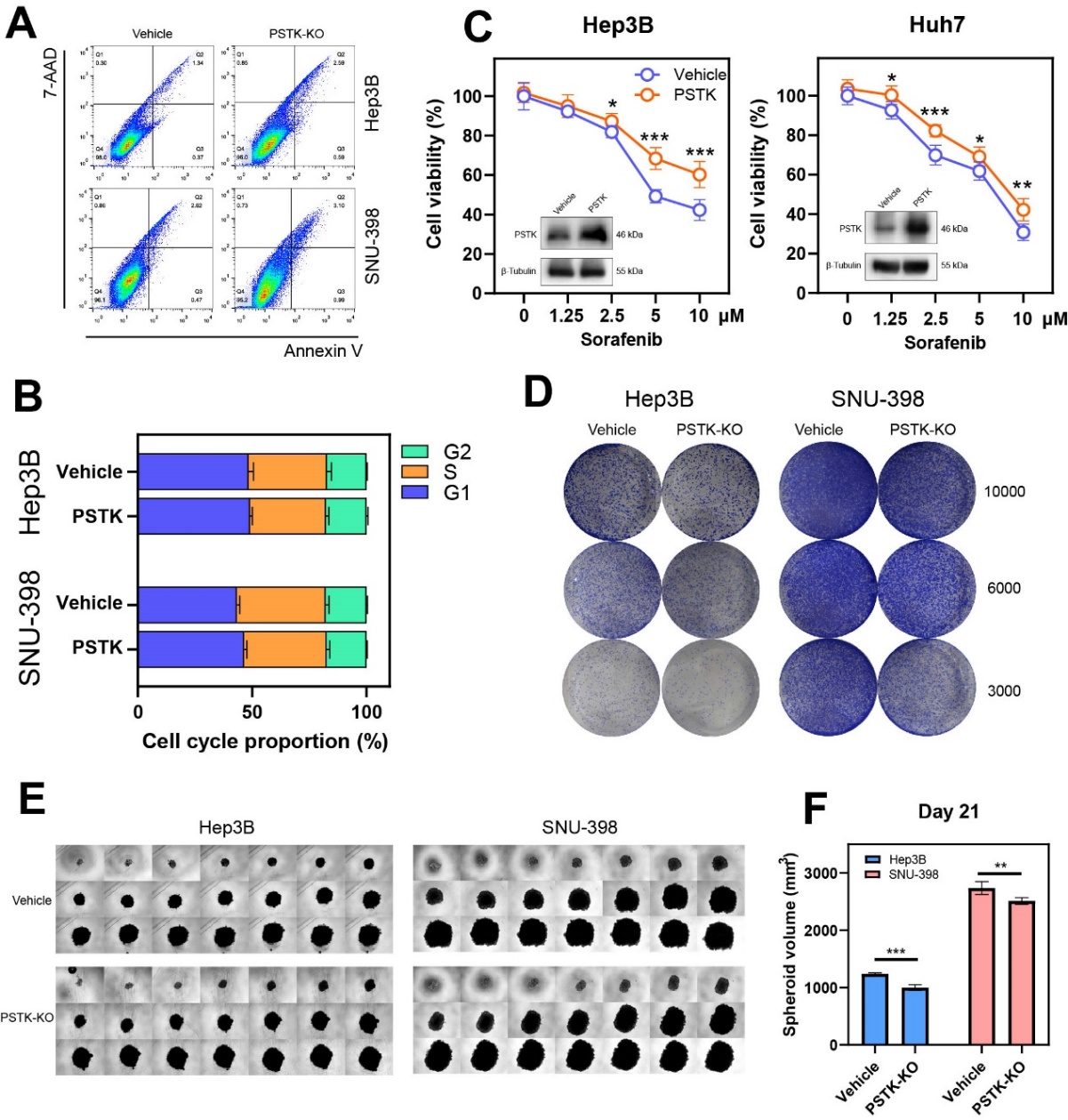


**Fig. S5. PSTK depletion induced a slight inhibition of proliferation in HCC cell lines.** **A.** Annexin V/7-AAD assay was used to investigate apoptosis rate in Hep3B-NC/PSTK-KO cells and SNU-398-NC/PSTK-KO cells (n=3). **B.** PI staining was used to investigate cell cycle proportion in Hep3B-NC/PSTK-KO cells and SNU-398-NC/PSTK-KO cells (n=3). **C.** Hep3B and Huh7 cells were transfected with PSTK expression plasmids and PSTK expression levels were confirmed by western blot. Then cells were treated with Sorafenib for 48 h, Cell viabilities were analyzed by CCK-8 assay. **D.** Long term growth assays of vehicle and PSTK-KO cells in 6-well plates for 10 days. **E.** Spheroid formation assay in ultra-low adherent round–bottom 96 well plates for 21 days (n=6). **F.** Spheroid volumes of vehicle and PSTK-KO cells in day 21. Student’s t test was used (*p<0.05, **p<0.01, ***p<0.001).


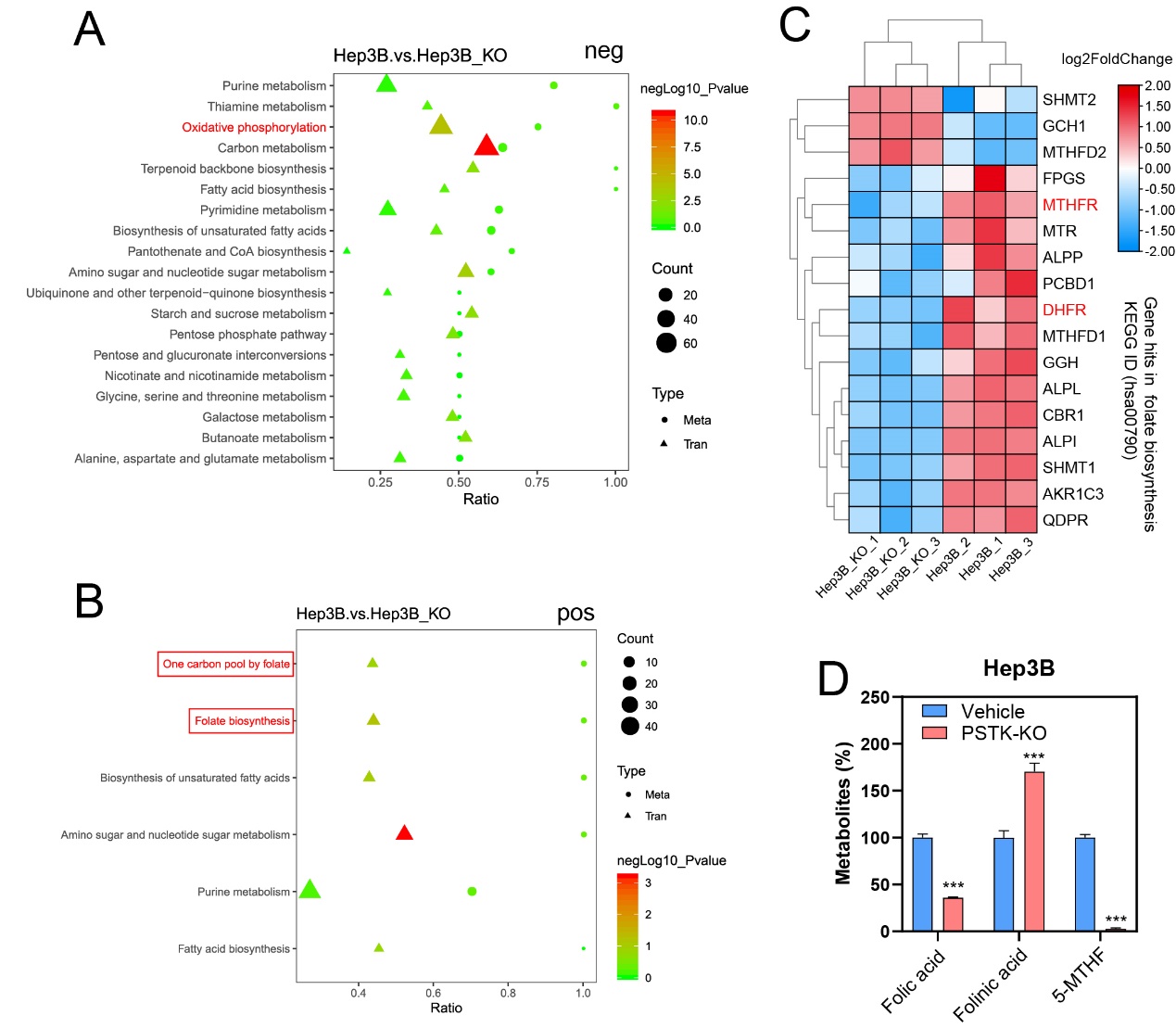


**Fig. S6. PSTK transcriptionally regulates folate biosynthesis process. A-B.** Correlation analyses of RNA-seq and metabolomics after PSTK knockout, KEGG enrichment analysis of common differentially expressed genes and metabolites. **C.** Heatmap displays the gene hits in the gene set of folate biosynthesis (KEGG ID: map00790). **D.** Typical metabolites of folate biosynthesis after PSTK knockout (5-MTHF: 5-Methyltetrahydrofolic acid). Student’s t test was used (***p<0.001).


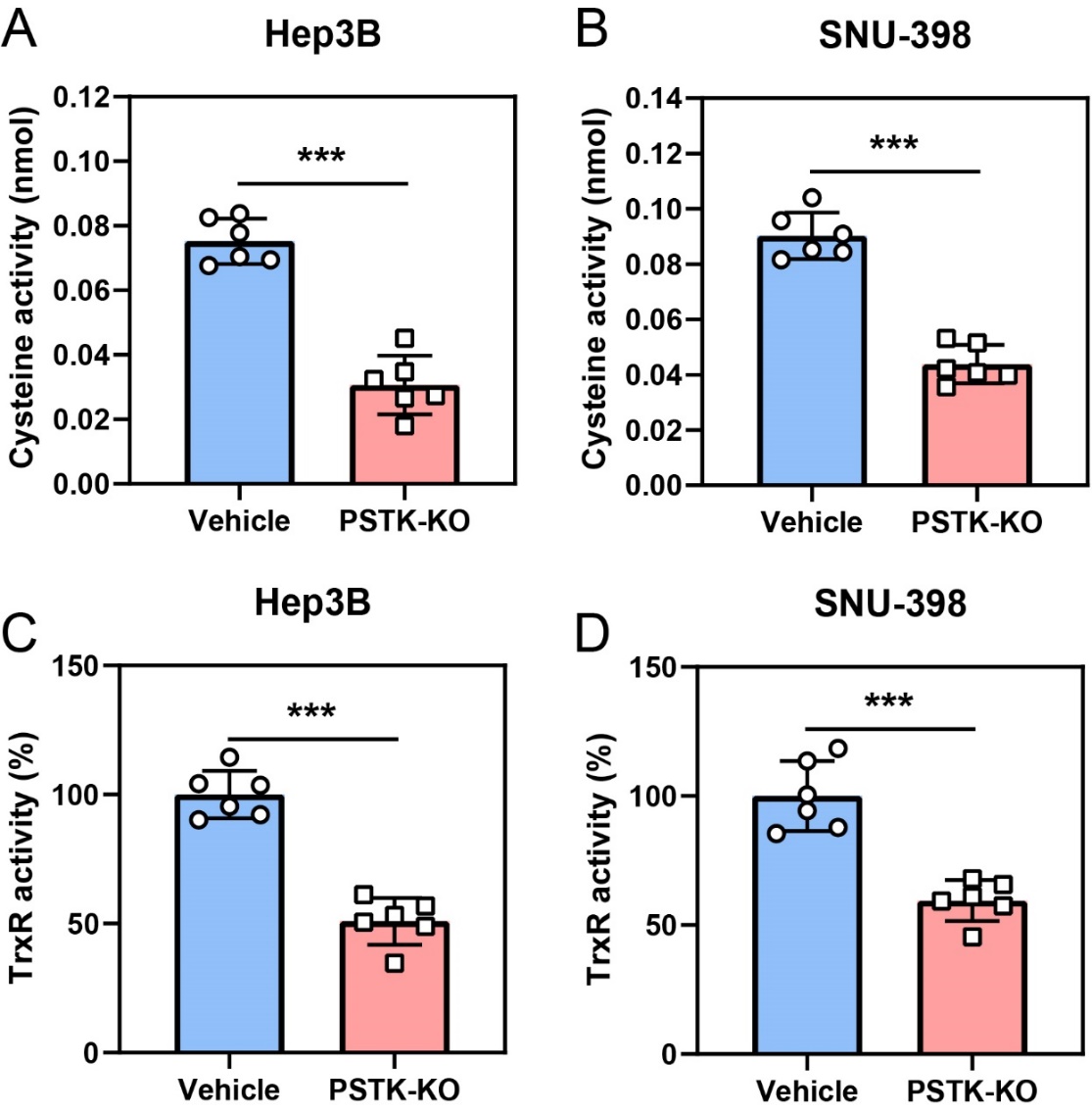


**Fig. S7. PSTK depletion induced deficiencies of cysteine synthesis and TxrR activity. A-B.** Cysteine concentrations were assayed in PSTK knockout cells and control cells (n=6). **C-D.** TrxR activities were assayed in PSTK knockout cells and control cells (n=6). Student’s t test was used (*** p<0.001).


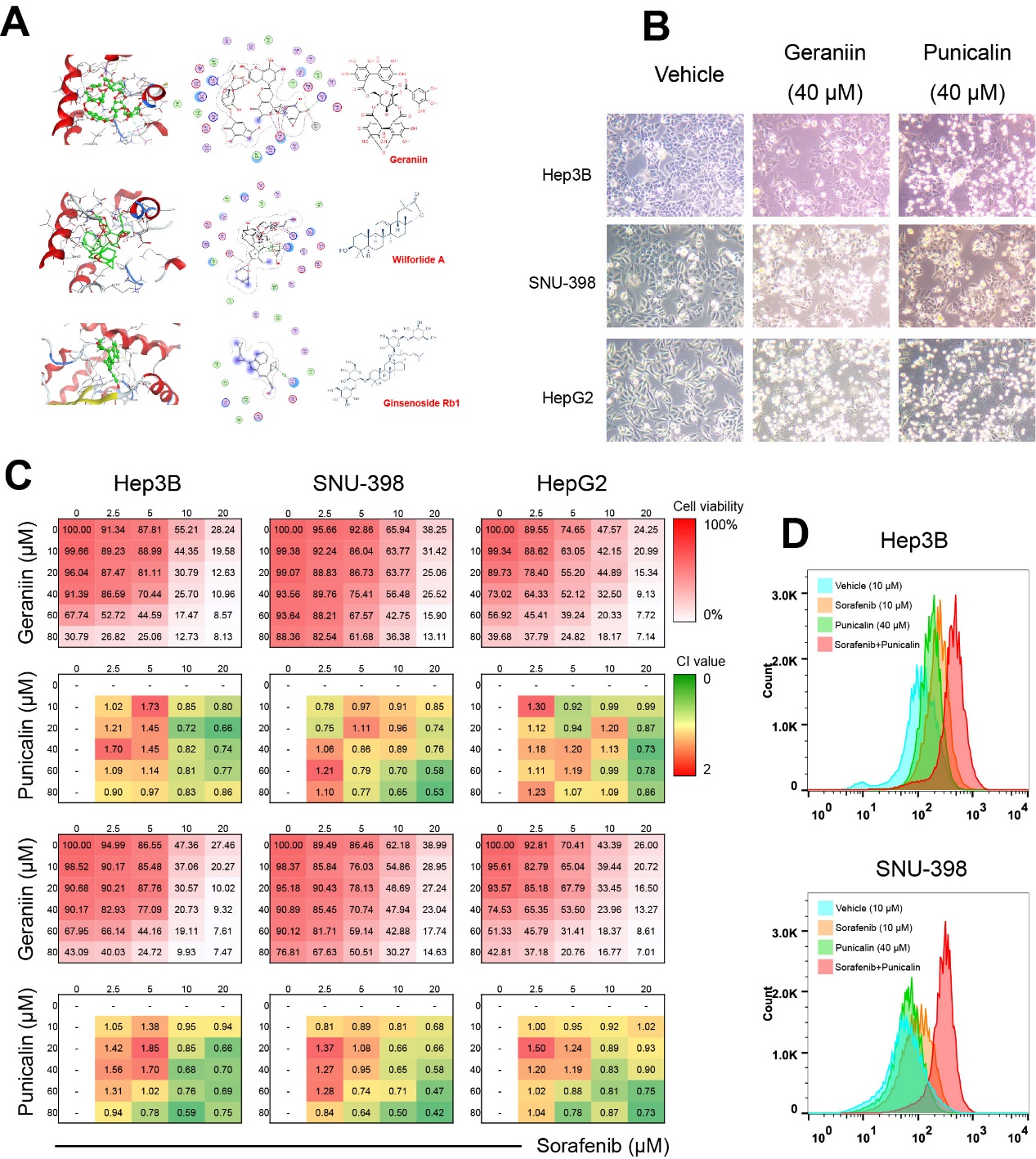


**Fig. S8 2D/3D modes of two potential PSTK inhibitors from virtual screen. A.** Geraniin binds to PSTK through three interactions, an electrostatic force with Asp41, an H-bonds with Met86 and an arene-H interaction with Lys200. Ginsenoside Rb1 contacts with Glu204、Asp79、Arg44 by ion interaction. H-bond can form with Lys196, and there is an arene-cation interaction with Lys200. Wilforlide A binds to Lys133 with arene-arene interaction. **B.** Geraniin and Punicalin showed significant anti-HCC effect. **C-D.** Punicalin/Geraniin exhibited synergy with Sorafenib when used to treat HCC cell lines in CCK-8 assay and ROS measurement**.**


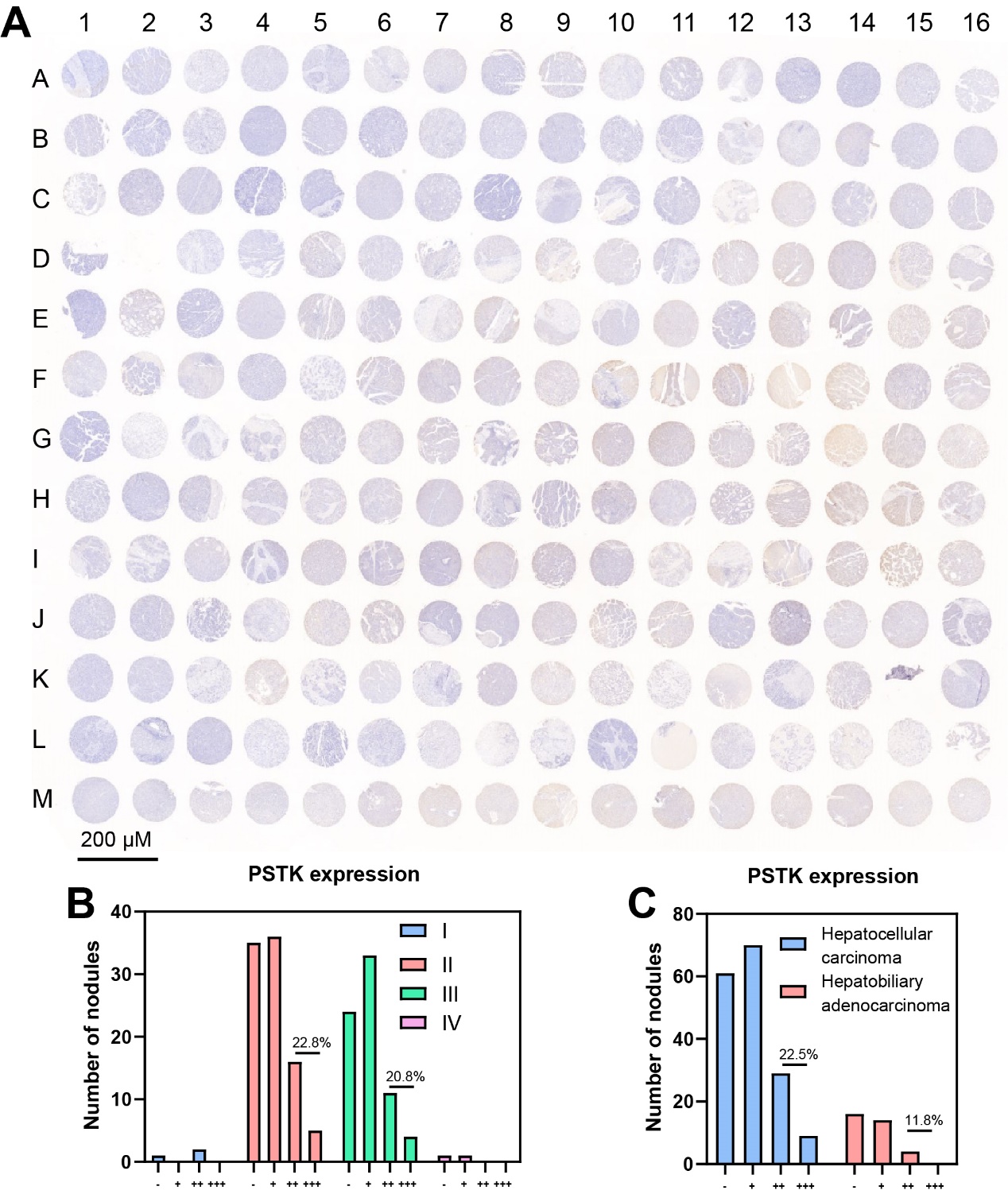


**Fig. S9. Panoramic scanning of PSTK staining tissue microarray containing 166 pathologically confirmed HCC samples. A.** Panoramic scanning figure. **B.** PSTK expression levels in different TNM stage HCC samples. **C.** Expression levels of PSTK in HCC and hepatobiliary adenocarcinoma.

**Supplementary tables**

**Table S1. Clinical characteristics of 50 patients with HCC**

| Variables | Expression  Low | Expression  High | χ2 p-value |
| --- | --- | --- | --- |
| Case (n) | 29 | 21 | NA |
| Age (Median, range) | 13/16 | 13/8 | 0.233 |
| Gender (male/female) | 23/6 | 17/4 | 0.886 |
| Tumor differentiation (I-II/III-IV) | 18/11 | 11/10 | 0.493 |
| Tumor size, cm (≤5/>5) | 20/9 | 15/6 | 0.851 |
| Tumor multiplicity (solitary/multiple) | 20/9 | 12/9 | 0.390 |
| Hepatitis (negative/positive) | 4/25 | 1/20 | 0.293 |
| Cirrhosis (absent/present) | 6/23 | 3/18 | 0.561 |
| AFP, ng/ml (≤25/>25) | 18/11 | 10/11 | 0.310 |
| Macrovascular invasion (absent/present) | 13/16 | 4/17 | 0.0575 |
| GPC3 (negative/positive) | 7/22 | 3/18 | 0.390 |
| Ki67+ (≤30%/>30%) | 10/19 | 9/12 | 0.547 |
| BCLC (A+B/C) | 14/15 | 4/17 | **0.0336** |
| TNM stage (I-II/III-IV) | 22/7 | 15/6 | 0.724 |

Abbreviation: AFP, a-fetoprotein; GPC3, Glypican-3; NA, not adopted, BCLC, Barcelona Clinic Liver Cancer.

Bold indicate significance of p value (p < 0.05).

**Table S2. Univariate analyses of factors associated with recurrence**

| Variables | Harzad Ratio (HR) | 95% CI | Univariate  p value |
| --- | --- | --- | --- |
| Age (median, range) |  |  | 0.711 |
| Gender (male/female) |  |  | 0.0521 |
| Tumor differentiation (I-II/III-IV) |  |  | 0.542 |
| Tumor size, cm (≤5/>5) |  |  | 0.330 |
| Tumor multiplicity (solitary/multiple) |  |  | 0.717 |
| Hepatitis (negative/positive) |  |  | 0.463 |
| Cirrhosis (absent/present) | 3.238 | 1.000-10.480 | **0.0499** |
| AFP, ng/ml (≤25/>25) |  |  | 0.176 |
| Macrovascular invasion (absent/present) |  |  | 0.910 |
| GPC3 (negative/positive) | 3.434 | 1.280-9.21 | **0.0164** |
| Ki67+ (≤30%/>30%) |  |  | 0.704 |
| BCLC (A+B/C) |  |  | 0.919 |
| TNM stage (I-II/III-IV) |  |  | 0.497 |
| PSTK (low/high) |  |  | 0.540 |
| GPX4 (low/high) |  |  | 0.429 |
| PSTK/GPX4 co-expression (low/high) | 2.541 | 0.901-7.170 | **0.0443** |

Abbreviation: AFP, a-fetoprotein; GPC3, Glypican-3; CI, confidence interval; NA, not adopted, BCLC, Barcelona Clinic Liver Cancer.

Bold indicate significance of p value (p < 0.05).

**Table S3. Primers for qPCR**

| Gene | Forward | Reverse |
| --- | --- | --- |
| TUBB | TGGACTCTGTTCGCTCAGGT | TGCCTCCTTCCGTACCACAT |
| GPX4 | GAGGCAAGACCGAAGTAAACTAC | CCGAACTGGTTACACGGGAA |
| FTH1 | CCCCCATTTGTGTGACTTCAT | GCCCGAGGCTTAGCTTTCATT |
| FTL | CAGCCTGGTCAATTTGTACCT | GCCAATTCGCGGAAGAAGTG |
| HMOX1 | AAGACTGCGTTCCTGCTCAAC | AAAGCCCTACAGCAACTGTCG |
| DHFR | CAGTAGAAGGTAAACAGAATCTGG | CCTTTAAAGGTCGATTCTTCTCAG |
| MTHFR | GAGCGGCATGAGAGACTCC | CCGGTCAAACCTTGAGATGAG |
| ALPI | TGAGGGTGTGGCTTACCAG | GATGGACGTGTAGGCTTTGCT |
| ALPL | ACCACCACGAGAGTGAACCA | CGTTGTCTGAGTACCAGTCCC |

**Reference**

1 Sellick, C. A., Hansen, R., Stephens, G. M., Goodacre, R. & Dickson, A. J. Metabolite extraction from suspension-cultured mammalian cells for global metabolite profiling. *Nature protocols* **6**, 1241-1249, doi:10.1038/nprot.2011.366 (2011).

2 Yuan, M., Breitkopf, S. B., Yang, X. & Asara, J. M. A positive/negative ion-switching, targeted mass spectrometry-based metabolomics platform for bodily fluids, cells, and fresh and fixed tissue. *Nature protocols* **7**, 872-881, doi:10.1038/nprot.2012.024 (2012).

3 Wen, B., Mei, Z., Zeng, C. & Liu, S. metaX: a flexible and comprehensive software for processing metabolomics data. *BMC bioinformatics* **18**, 183, doi:10.1186/s12859-017-1579-y (2017).

4 Trott, O. & Olson, A. J. AutoDock Vina: improving the speed and accuracy of docking with a new scoring function, efficient optimization, and multithreading. *Journal of computational chemistry* **31**, 455-461, doi:10.1002/jcc.21334 (2010).

5 Ashton, J. C. Drug combination studies and their synergy quantification using the Chou-Talalay method--letter. *Cancer research* **75**, 2400, doi:10.1158/0008-5472.Can-14-3763 (2015).
